# Detection of a chemical cue from the host seaweed *Laurencia dendroidea* by the associated mollusc *Aplysia brasiliana*

**DOI:** 10.1371/journal.pone.0187126

**Published:** 2017-11-02

**Authors:** N. Nocchi, A. R. Soares, M. L. Souto, J. J. Fernández, M. N. Martin, R. C. Pereira

**Affiliations:** 1 Programa de Pós-graduação em Dinâmica do Oceano e da Terra, Universidade Federal Fluminense, Campus da Praia Vermelha, Niterói, Brazil; 2 Grupo de Produtos Narturais de Organismos Aquáticos (GPNOA), Universidade Federal do Rio de Janeiro, Núcleo em Ecologia e Desenvolvimento Sócio-Ambiental de Macaé, Macaé, Brazil; 3 Instituto Universitario de Bio-Orgánica “Antonio González” (IUBO), Centro de Investigaciones Biomédicas de Canarias (CIBICAN), Departamento de Química Orgánica, Universidad de La Laguna (ULL), La Laguna, Tenerife, España; Texas A&M University Corpus Christi, UNITED STATES

## Abstract

Chemical cues from sessile hosts can attract mobile and associated organisms and they are also impotant to maintain associations and overall biodiversity, but the identity and molecular structures of these chemicals have been little explored in the marine environment. Secondary metabolites are recognized as possible chemical mediators in the association between species of *Laurencia* and *Aplysia*, but the identity of the compounds has not been established. Here, for the first time, we experimentally verify that the sesquiterpene (+)-elatol, a compound produced by the red seaweed *Laurencia dendroidea*, is a chemical cue attracting the associated sea hare *Aplysia brasiliana*. In addition to revealing the nature of the chemical mediation between these two species, we provide evidence of a chemical cue that allows young individuals of *A*. *brasiliana* to live in association with *L*. *dendroidea*. This study highlights the importance of chemical cues in *Laurencia*-*Aplysia* association.

## Introduction

In the marine environment, chemical cues or waterborne infochemicals act as mediators in different intra- and inter-specific ecological interactions between organisms [1], such as chemical defense [2], settlement and recruitment of larvae [3], feeding stimuli [4], foraging behavior [5], selection and localization of prey [6], among others. Marine organisms produce a wide variety of molecules that mediate these ecological interactions [1, 7]. Despite their broad and crucial roles in population structure, community organization and ecosystem function in marine systems, the omnipresence of these chemical cues and their impacts are still inadequately recognized [1]. However, scientific efforts have been increasing to elucidate the mechanisms of chemical communication between organisms in marine environments [8].

Red seaweeds of the genus *Laurencia* are well established as rich producers of secondary metabolites [9]. Since investigations of this taxon began more than 60 years ago, more than 1,000 secondary metabolites such as terpenes and C_15_ acetogenins (both halogenated or not) have been isolated from about 60 species [10]. These metabolites not only possess unique structural features, but they also exhibit significant ecological roles as antifoulants [11] and as a defense against herbivores [12]. Despite the strong defensive properties of these chemicals, *Aplysia* sea hares selectively graze on *Laurencia* species and have the ability to sequester and store secondary metabolites from these red seaweeds in a specialized digestive gland [13,14]. A huge number of molecules have been discovered from *Aplysia* species [15] and these sea hares possess secondary metabolites obtained either from their algal diets or by *de novo* direct synthesis [16], with the former being slightly modified. This feeding relationship makes *Aplysia* species promoters of chemical diversity, since most of the metabolites found in these molluscs are structurally similar, but distinct from those produced by *Laurencia* species [17].

The maintenance of the relationship between *Laurencia* and *Aplysia* species is probably due to the adaptative evolution of these herbivores to metabolism of the seaweed chemicals giving them the ability to use as a self-defense mechanism [18,19]. In addition, some photosynthetic proteins from *Laurencia* are converted to an effective chemical defense mechanism displayed by *Aplysia*, which releases them as a purple ink and opaline when attacked by predators [20]. In fact, these secretions contain vital metabolites for *Aplysia* species, which can act as feeding stimulants, feeding deterrents, and aversive chemicals [21].

In general, host seaweeds are preferred sites for metamorphosis of larvae in most species of sea hares [22]. For *Aplysia* species we observed that these organisms preferentially inhabit and grow better on red seaweeds, including *Laurencia* species [13,14]. Evidence has shown that chemical mediation is vital in interactions between species of *Laurencia* and *Aplysia* [17], but the nature of the compounds involved in this interaction has not been established [7]. Although *Aplysia* and *Laurencia* are rich sources of secondary metabolites, these compounds have been mainly studied in the context of bioprospecting for bioactive molecules [17,23].

Due to the close and chemically-mediated relationship between *Laurencia* algae and *Aplysia* sea hares, we hypothesized that secondary metabolites produced by *Laurencia dendroidea* can act as important chemical cues for the specialist predator *Aplysia brasiliana*. In this work, we addressed the following two questions, evaluated under laboratory conditions: (1) Which compounds in seawater are exudates from *L*. *dendroidea*? and (2) How does the mollusc *A*. *brasiliana* respond to *L*. *dendroidea* metabolites in bioassays? Although many studies assume its occurrence, to our knowledge this is the first study that tested and identified *Laurencia* chemical cues for *Aplysia*.

## Materials and methods

### Collection of organisms

Specimens of *Laurencia dendroidea* and young individuals (4.8 ± 1.5 SD cm in length; N = 194) of the sea hare *Aplysia brasiliana* found living in association with these algae were collected from the intertidal zone of Azeda Beach (Búzios, 22°44'33.6'' S, 41°52'55.6'' W, Rio de Janeiro State, Brazil) by free diving in September, 2014. After collection, all the organisms were transported to the laboratory in individual aquaria containing seawater with aeration and maintained under constant temperature (22°C), aeration, salinity (35), and a 12 h:12 h light:dark photoperiod. During the acclimatization period of 48 hours, the specimens of *A*. *brasiliana* were fed with fresh palatable green seaweed *Ulva* sp. before starting the assays.

Collection of organisms was supported by authorization SISBIO 34321–1 (Instituto Chico Mendes de Conservação da Biodiversidade).

### *Laurencia-*conditioned seawater (LCW) and *Laurencia*-exuded metabolites in seawater extract (SEM)

For the laboratory detection of the *L*. *dendroidea* exuded metabolites in seawater, a proportion of 24 g of algae mass to 1 L of artificial seawater (“Sea salts” SIGMA, USA, in distilled water) was used for incubation in fifteen Erlenmeyer flasks of 3 liters each. They were kept for 12 hours in a BOD chamber at 20°C, under constant ilumination and constant aeration. Then, the algae were taken out from the flasks and the conditioned water was passed through a 0.45 μm filter Millipore system to yield the *Laurencia*-conditioned seawater (LCW). Part of this LCW was used immediately in Y-maze tests (20 L), and the remaining LCW (10 L) was used to identify the metabolites exuded by *L*. *dendroidea*. Artificial seawater (without the presence of *L*. *dendroidea*) was used as a control for chemical analyses and as a control stimulus in the Y-maze tests. The portion of the LCW separated for chemical analysis was passed through a glass column filled with DIAON HP20 resin (Sigma-Aldrich). Then the resin was washed with ultrapure water to eliminate salts and the metabolites retained in the resin were eluted with 600 mL of methanol (Tedia, HPLC), that was evaporated to yield the *Laurencia*-exuded metabolites in seawater extract (SEM). The same procedure was used to produce a representative control from artificial seawater. Both the SEM and the control extract from artificial seawater were analyzed by GC/MS.

### Chemical extraction and identification of secondary metabolites

Whole fresh tissues of *L*. *dendroidea* (450 g) were extracted in EtOAc/MeOH (1:1 v/v). The resulting extract was filtered and the solvent was eliminated in a rotary evaporator under reduced pressure. The extract (1.4 g) was submited to size exclusion chromatography using Sephadex LH-20 (6 x 34 cm, Pharmacia Fine Chemicals^®^) and eluted with *n-*hexane/CH_2_Cl_2_/MeOH (2:1:1). Seven fractions (FR_A_ to FR_G_) were obtained and analyzed by Nuclear Magnetic Resonance (NMR) of ^1^H. From the ^1^H NMR analysis the fraction-enriched with sesquiterpenes (283 mg, FR_D_) was subsequently processed by chromatography in medium-pressure Lobar LiChroprep Si-60 (40–63 μm, MERCK^®^) using *n*-hexane/EtOAc (9:1) at 2 mL min^-1^. Twelve fractions (FR_1_ to FR_12_) were obtained, which allowed isolation of compounds **1 (**1.45 mg, FR_4_**)** and **2** (120.49 mg, FR_5_). Fractions FR_7_ and FR_8_ were purified using high-performance liquid chromatography (HPLC) with a μ-Porasil^™^ silica column (125 Å; 1.9 x 15 cm) and eluted with *n*-hexane/EtOAc (9:1), which yielded compounds **3** (0.52 mg) and **4** (1.51 mg) from fraction FR_8_ and compound **5** (2.7 mg) from fraction FR_7_.

The chemical structures of the isolated compounds were established by NMR of ^1^H and ^13^C, correlation spectroscopy (COSY), heteronuclear multiple-bond correlation (HMBC) and heteronuclear single quantum correlation (HSQC) in a Bruker^®^ Avance 600 MHz system using CDCl_3_. Chemical shifts were reported in ppm referenced to solvent signals (CDCl_3_: δ_H_ 7.26, δ_C_ 77.16). Mass spectrometric data were acquired using a VG-Autospec Fisons system in electrospray ionization mode. The specific rotations [α]_D_ were determined using a Polarimeter Perkin-Elmer^®^ (model 241), under conditions of 20°C, sodium D line λ = 589 nm and cell path length 1 dm. All data were compared with the available literature [24–29].

*10-bromo-9-hydroxy-chamigra-2*,*7(14)-diene* **(1)**: oil; [α]_D_ -125 (*c* 0.008, CHCl_3_), lit. [α]_D_ -110 (c 0.20, CHCl_3_) [24]; ESI-HRMS (*m/z*): [M+Na]^+^ calcd. for C_15_H_23_^79^BrONa, 321.0830 and C_15_H_23_^81^BrONa, 323.0810; found 321.0834 and 323.0807 (ratio 100:97), respectively. All other physical and spectroscopic data are in agreement with those previously reported [24].

*(+)-elatol* **(2)**: oil; [α]_D_ +79 (*c* 0.06, CHCl_3_), lit. [α]_D_ +75 (*c* 1.01, CHCl_3_) [24,25]; ESI-HRMS (*m/z*): [M+Na]^+^ calcd. for C_15_H_22_O^35^Cl^79^BrNa, 355.0440 and C_15_H_22_O^35^Cl^81^BrNa, 357.0420; found 355.0443 and 357.0435 (ratio 100:94), respectively. All other physical and spectral properties are in agreement with published data [25,26].

*(Z)-10*,*15-dibromo-9-hydroxychamigra-1*,*3(15)*,*7(14)-triene* **(3)**: colorless oil; [α]_D_ +10 (*c* 0.02, CHCl_3_), lit. [α]_D_ +3 (*c* 0.002, CHCl_3_) [26]; ESI-HRMS (*m/z*): [M+Na]^+^ calcd. for C_15_H_20_O^79^Br_2_Na, 396.9779; C_15_H_20_O^79^Br^81^BrNa, 398.9758 and C_15_H_20_O^81^Br_2_Na, 400.9738; found 396.9779, 398.9756, and 400.9749 (ratio 48:100:52), respectively. All other physical and spectroscopic data are comparable to those previously reported [27].

*(E)-10*,*15-dibromo-9-hydroxychamigra-1*,*3(15)*,*7(14)-triene* **(4)**: colorless oil; [α]_D_ -31 (*c* 0.016, CHCl_3_), lit. [α]_D_ -40 (*c* 0.01, CHCl_3_) [26]; ESI-HRMS (*m/z*): [M+Na]^+^ calcd. for C_15_H_20_O^79^Br_2_Na, 396.9779, C_15_H_20_O^79^Br^81^BrNa, 398.9758 and C_15_H_20_O^81^Br_2_Na, 400.9738; found 396.9784, 398.9761, and 400.9756 (ratio 48:100:52), respectively. All other physical and spectroscopic data are in agreement with those previously reported [27].

*isoobtusol* **(5)**: white crystal; [α]_D_ +77 (*c* 0.03, CHCl_3_), lit. [α]_D_ +25 (*c* 0.50, CHCl_3_) [27–28]; ESI-HRMS (*m/z*): [M+Na]^+^ calcd. for C_15_H_23_^79^Br^81^Br^35^ClONa, 436.9681, C_15_H_23_^79^Br_2_^35^ClONa, 436.9672 and C_15_H_23_^81^Br_2_^35^ClONa, 438.9661; 434.9732, 436.9694, and 438.9674 (ratio 48:100:73), respectively. All other physical and spectroscopic data are in agreement with those previously reported [27–29].

### GC/MS analysis

Chemical profiles were obtained on a GC/MS Agilent 7890B, MS7000C (using an automatic injector CombiPAL with the control software MassHunter) in the electron impact mode (70 eV). Chromatographic separation was achieved with a HP-5MS UL capillary column (15 m x 0.25 mm x 0.25 μm). Helium was used as the carrier gas (flow rate of 1 mL min^-1^). The oven temperature was 60°C for the first 2.5 min, then increased at a rate of 10°C.min^-1^ to 280°C and held at the latter temperature for 6 min, with a total run time of 30.5 min. Injector and ion source temperatures were 300 and 240°C. Samples were injected in splitless mode. The detection was performed in the full scan mode, using a mass range of 60–400 *m/z*. All samples were solubilized in dichloromethane and filtered through a PTFE Syringe filter (pore width of 0.45μm; diameter of 13mm) before the injections.

**Fig 1:**
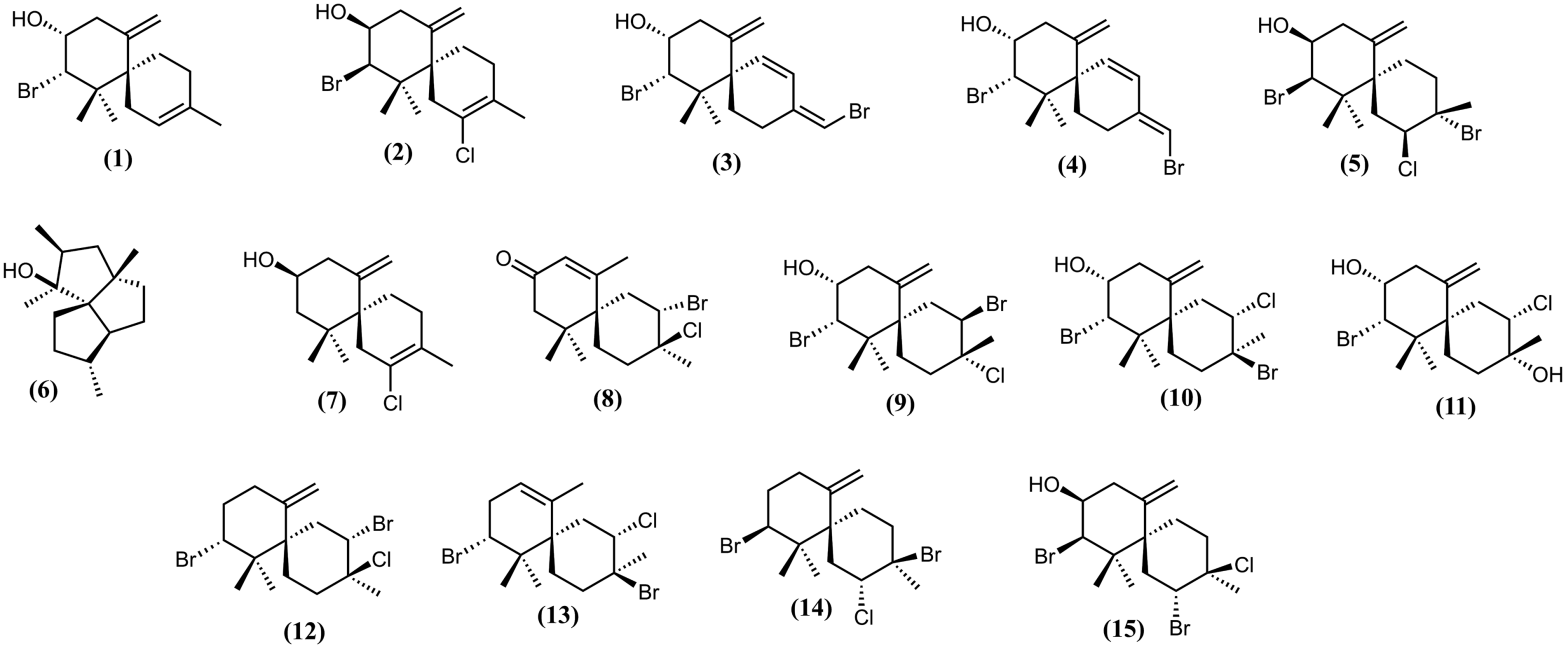
Chemical structures of the metabolites found in *L*. *dendroidea*.

The chemical profiles of all the extracts (whole algal extract, SEM and control) were obtained by GC/MS. The isolated compounds (**1**–**5**) and purified standards (**6**–**15**) (Fig 1) available in our laboratory were also analyzed by GC/MS to construct a *L*. *dendroidea* metabolites database. Compounds **6**–**15** were obtained as described by the literature [30–33]. Identification of metabolites exuded into seawater by *L*. *dendroidea* (SEM) was performed by comparing the peaks of the SEM chromatogram with its respective control (artificial seawater extract). A baseline alignment of each chromatogram was performed prior to comparison and the time-shift among peaks was corrected by the Correlation Warping Algorithm (COW), as described by Nielsen and collaborators (1998) [34], using the software COW (http://www2.biocentrum.dtu.dk/mycology/analysis/cow). Compounds present in the algal extract, and SEM extract were identified based on their mass spectra data, retention time, and comparison with pure compounds available in our laboratory.

The compound identified from the SEM extract (elatol) was quantified using external standardization by means of an analytical curve calculated from four different concentrations of purified elatol in dichloromethane (10 to 10^−2^ μg mL^-1^). Detection and quantification limits were determined according to United States Environmental Protection Agency provisions; we obtained an analytical curve in a concentration range almost 5 times the noise of the baseline. Then, the concentrations of three replicates of the standard were determined from this curve, and its standard deviation was calculated. The relative standard deviation (RSD) or coefficient of variation (CV) was 10%.

### Y-maze experiments

**Fig 2:**
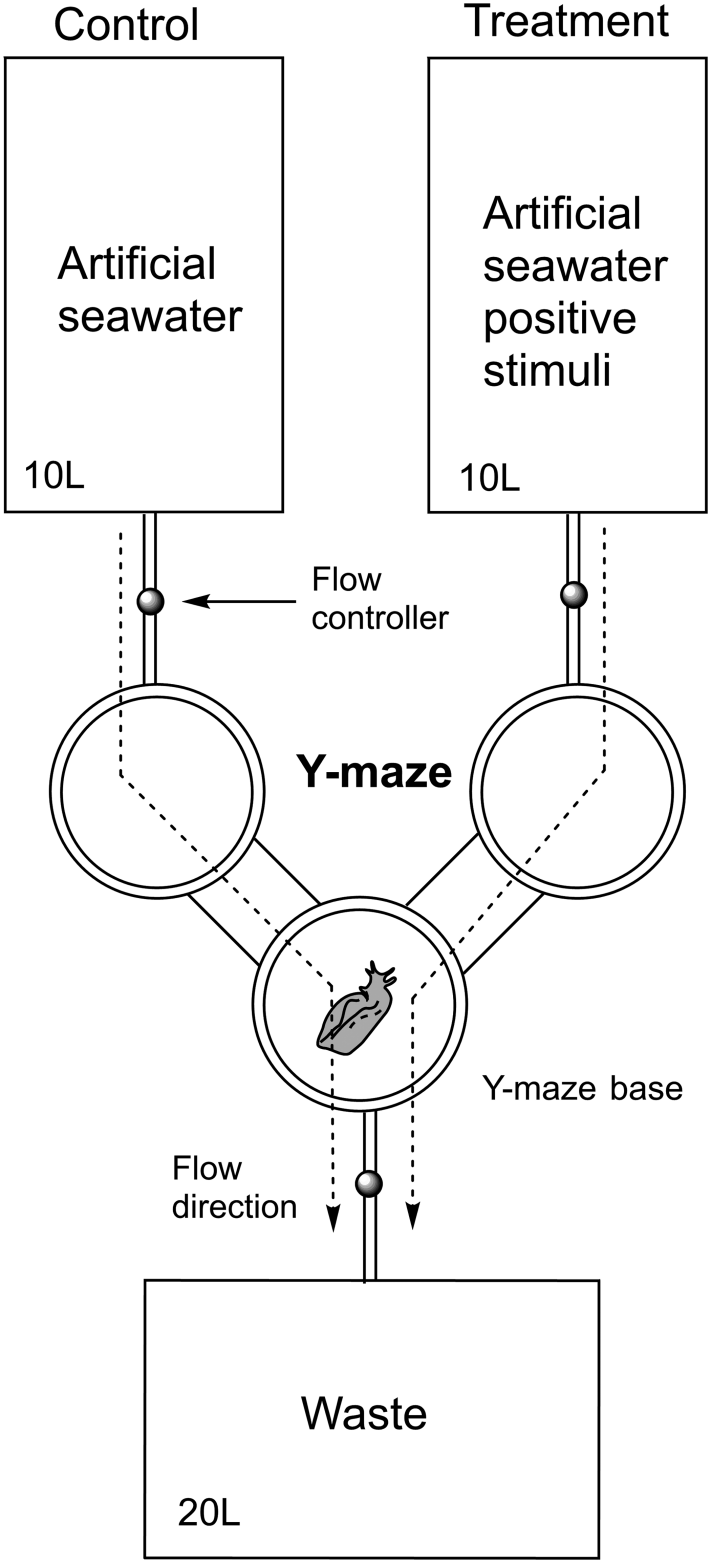
The maze used in this study. The control was in the left aquarium flowing pure seawater and the treatment was in the right aquarium flowing seawater plus a stimuli (e.g. *Laurencia* tallus *or Laurencia*-exuded metabolites in seawater extract (SEM).

Y-maze behavioural bioassays were performed to determine whether the specimens of *A*. *brasiliana* would be attracted by *L*. *dendroidea* compounds. The maze used in this study consisted of a Y-shaped plastic tube connected to two 10 L aquariums, with seawater flowing by gravity from the aquariums through each branch of the Y-maze (Fig 2). One of the aquariums contained artificial seawater with the tested stimuli (treatment), while the other was filled with pure artificial seawater, i.e. the control (see below). Water flowed from the two aquariums (treatment and control) through the Y-maze and exited at the base of the conjoined branch. Soluble coloring was used to verify the water plume and flow regulators were used to standardize the flux in the control and treatment aquaria before each experiment. The artificial seawater was used at 26°C and a salinity of 35.

In the intervals between each assay, the entire Y-maze was washed with distilled water and rinsed with seawater and control and stimulus arms of the Y-maze were switched. The seawater flow in each branch of the Y-maze was controlled by flow controllers. Individuals of *A*. *brasiliana* were initially positioned at the base of the Y-maze at the start of each bioassay.

Six experiments were undertaken with individual *A*. *brasiliana* choosing between stimulus *vs* artificial seawater. Each experiment consisted to introduce one sea hare at the base of the Y-maze and its behavior in terms of choosing between the arms containing either the stimulus or the artificial seawater was observed for up to 15 min. Three responses were considered: (a) positive, when *A*. *brasiliana* swam to the end of the stimulus arm; (b) negative, when *A*. *brasiliana* swam to the end of the control arm; and (c) no response, when *A*. *brasiliana* did not choose either one of the arms within the 15 min time frame. The stimulus tested in each experiment was drive in the follow sequence: 1) Control stimulus (n = 21): both arms of the Y-maze tube were filled only with artificial seawater in order to verify the ability of *A*. *brasiliana* individuals to overcome the water flow in the branches of the Y-maze structure; 2) Fresh *L*. *dendroidea* (n = 32): one specimen of this seaweed was placed in the stimulus arm of the Y-maze, while a plastic *L*. *dendroidea* mimic with equivalent size and volume of this fresh seaweed was placed in the artificial seawater arm (control) to maintain an equivalent flow of water through the tube during the experiment; 3) *Laurencia-*conditioned seawater (LCW): the stimulus aquarium was filled with LCW while only artificial seawater was used in the control tank. A total of 43 replicates were used; 4) *L*. *dendroidea* extract: the effect of compounds obtained from fresh *L*. *dendroidea* on *A*. *brasiliana* was evaluated using the extract at its obtained concentration (the amount of extract per algae dry mass, 146 mg g^-1^). A piece of filter paper was cut into squares 1.5 × 1.5 cm and each square was equivalently soaked into the extract previously dissolved in dichloromethane (concentration of extract determined as the extract equivalent to the dry weight of alga = dry weight of filter paper). For the control, an equivalent square piece of paper was soaked in dichloromethane without the extract. After evaporating the solvent, each piece of paper of control and treatment was positionated at its respective Y-maze arm. A total of 42 replicates was used; 5) *Laurencia*-exuded metabolites in seawater extract (SEM): To evaluate the effect of SEM extract (using the obtained concentration of extract per algal dry mass incubated, 5 mg g^-1^) on the *Aplysia* specimens, the experiments were conducted in the same way as described for LCW above. A total of 30 replicates was used; and 6) Elatol-conditioned water (ECW): the effect of elatol (**2**), the compound identified in the SEM by GC/MS, on *Aplysia* specimens was investigated. The amount of treatment added was such that the final concentration was equal to the natural isolated concentration based on seawater volume used for extraction (0.02 mg L^-1^). The compound was solubilized in 35μL DMSO and added to the artificial seawater in the stimulus arm. The same amount of 35μL DMSO was added to the artificial seawater in the control arm. A total of 26 replicates were used in this experiment. In the intervals between each assay, the entire Y-maze was washed and the control and stimulus arms of the Y-maze were switched in order to remove any remaining *A*. *brasiliana* scents or previous chemical cues. For each assay a new *A*. *brasiliana* specimen was used. In the end of the experiments all the animals were returned to field.

The significance of *A*. *brasiliana* behavioral choice was evaluated by a chi-squared test that compared the number of times that *A*. *brasiliana* moved to for the three response groups (positive response, negative resposnde and no response) against an equal random distribution. Differences between the groups were deemed statistically significant when p ≤ 0.05.

## Results

### GC/MS database of metabolites of *Laurencia dendroidea*

A total of 15 sesquiterpenes of *L*. *dendroidea* were analysed by gas chromatography/mass spectrometry (GC/MS) in order to obtain a GC/MS database of *Laurencia* metabolites (supp 1). Extract of *L*. *dendroidea* yielded compounds **1**–**5**, which were purified using a series of chromatographic steps over Sephadex LH-20, silica gel and normal-phase high performance liquid chromatography (HPLC). The compounds were elucidated based on spectral data: optical rotation, infrared, electron ionization (EIMS) and/or high-resolution electron-impact mass spectroscopy (HREIMS), 1D and 2D NMR. The known halogenated chamigrane sesquiterpenes were identified by comparing their spectroscopic data with the literature as follows: 10-bromo-9-hydroxy-chamigra-2,7(14)-diene **(1)** [24], (+)-elatol **(2)** [25], (*Z*)-10,15-dibromo-9-hydroxy-chamigra-1,3(15),7(14)-triene **(3)** [27], (*E*)-10,15-dibromo-9-hydroxy-chamigra-1,3(15),7(14)-triene **(4)** [27] and isoobtusol **(5)** [28] (The NMR data and all the MS and NMR spectra of **1**–**5** are available in the supplemental material). Another 10 metabolites that were isolated previously by our research group [30–33] were used here to further populate the GC/MS *Laurencia* metabolite database: triquinane **(6)** [31,33]; debromoelatol **(7)** [30,31]; enone of desbromocartilagineol or laurencenone D **(8)** [35]; cartilagineol **(9)** [30,31]; obtusol **(10)** [31–32]; dendroidiol **(11)** [30,31]; nidificene **(12)** [31]; 3,10-dibromo-4-chloro-alpha-chamigrane **(13)** [31]; (+)-obtusane **(14)** [30] and rogiolol **(15)** [31].

### Identification of metabolites in extracts of *Laurencia dendroidea* by GC/MS analysis

**Fig 3:**
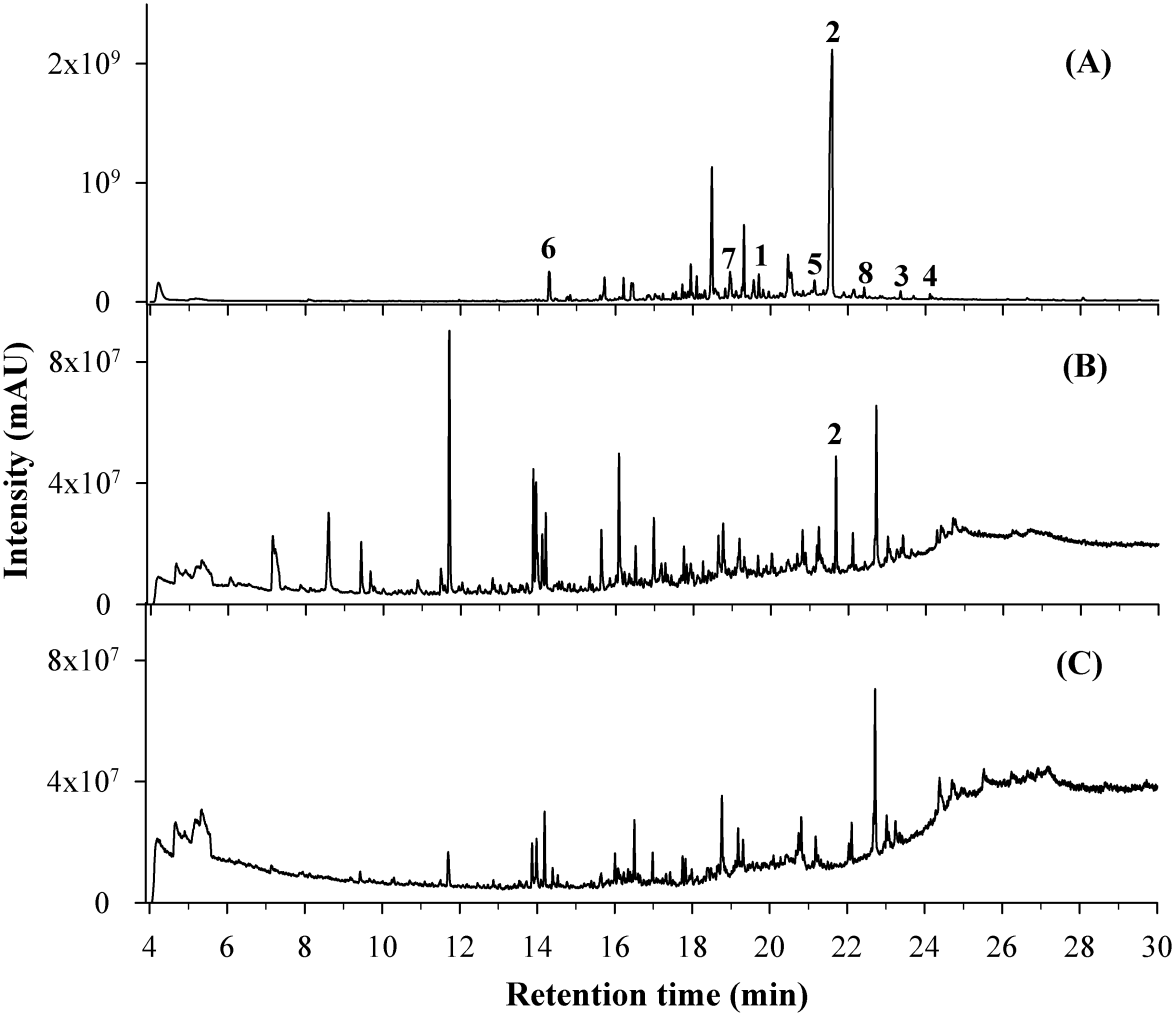
GC/MS chromatograms. **(A)** *L*. *dendroidea* extract. **(B)** *Laurencia*-exuded metabolites in seawater extract (SEM). **(C)** Control extract (from artificial seawater). Compounds identified using the GC/MS *Laurencia* metabolite database: 10-bromo-9-hydroxy-chamigra-2,7(14)-diene **(1)**, (+)-elatol **(2)**, (*Z*)-10,15-dibromo-9-hydroxy-chamigra-1,3(15),7(14)-triene **(3)**, (*E*)-10,15-dibromo-9-hydroxy-chamigra-1,3(15),7(14)-triene **(4)**, isoobtusol **(5)**, triquinane **(6)**, debromoelatol **(7)** and enone of desbromocartilagineol or laurencenone D **(8)**.

The *L*. *dendroidea* extract, *Laurencia*-exuded metabolites in seawater extract (SEM) and control extract (extract from artificial seawater) were analyzed by GC/MS. The retention times and mass spectroscopy patterns of the compounds in these extracts were compared to the *L*. *dendroidea* metabolites database (Table 1 and Fig 3). Compounds **1**–**8** were detected in the *L*. *dendroidea* extract (Fig 3A), whereas only compound **2** (elatol) was found in the exudate (SEM) (Fig 3B) at a concentration of 0.02 mg L^-1^ (or 0.0007 mg g^-1^ by algal wet weight; y = 1E+10x-3E+08; R^2^ = 0.99). No metabolites were identified in the control extract (Fig 3C).

**Table 1:** Retetion time (r_t_); main ions (*m/z*) observed in the mass spectra (EIMS) and detection of sesquiterpenes in the *L*. *dendroidea* extract and *Laurencia*-exuded metabolites in seawater extract (SEM), as characterized by GC/MS analysis. The presence of identified compounds in the samples was marked (x).

r_t_ (min)	*L*. *dendroidea* compounds database	*m/z*	*L*. *dendroidea* extract	SEM
14.48	triquinane **(6)**	222; 204;135	x	
18.71	debromoelatol **(7)**	236; 221; 208	x	
19.38	cartilagineol **(9)**	317; 299; 235		
19.74	10-bromo-9-hydroxy-chamigra-2,7(14)-diene **(1)**	283; 201; 269	x	
21.57	isoobtusol **(5)**	317; 299; 235	x	
21.76	(+)-elatol **(2)**	299; 253; 199	x	x
22.25	enone of desbromocartilagineol or laurencenone D **(8)**	278; 199; 161	x	
22.49	dendroidiol **(11)**	332; 299; 235		
23.09	nidificene **(12)**	396; 316; 237		
23.09	3,10-dibromo-4-chloro-alpha-chamigrane **(13)**	396; 316; 237		
23.15	(+)-obtusane **(14)**	396; 316; 237		
23.38	(*Z*)-10,15-dibromo-9-hydroxy-chamigra-1,3(15),7(14)-triene **(3)**	296; 240; 279	x	
23.55	(*E*)-10,15-dibromo-9-hydroxy-chamigra-1,3(15),7(14)-triene **(4)**	296; 240; 279	x	
24.48	rogiolol **(15)**	317; 299; 235		
24.55	obtusol **(10)**	317; 299; 235		

### Y-maze bioassays

**Fig 4:**
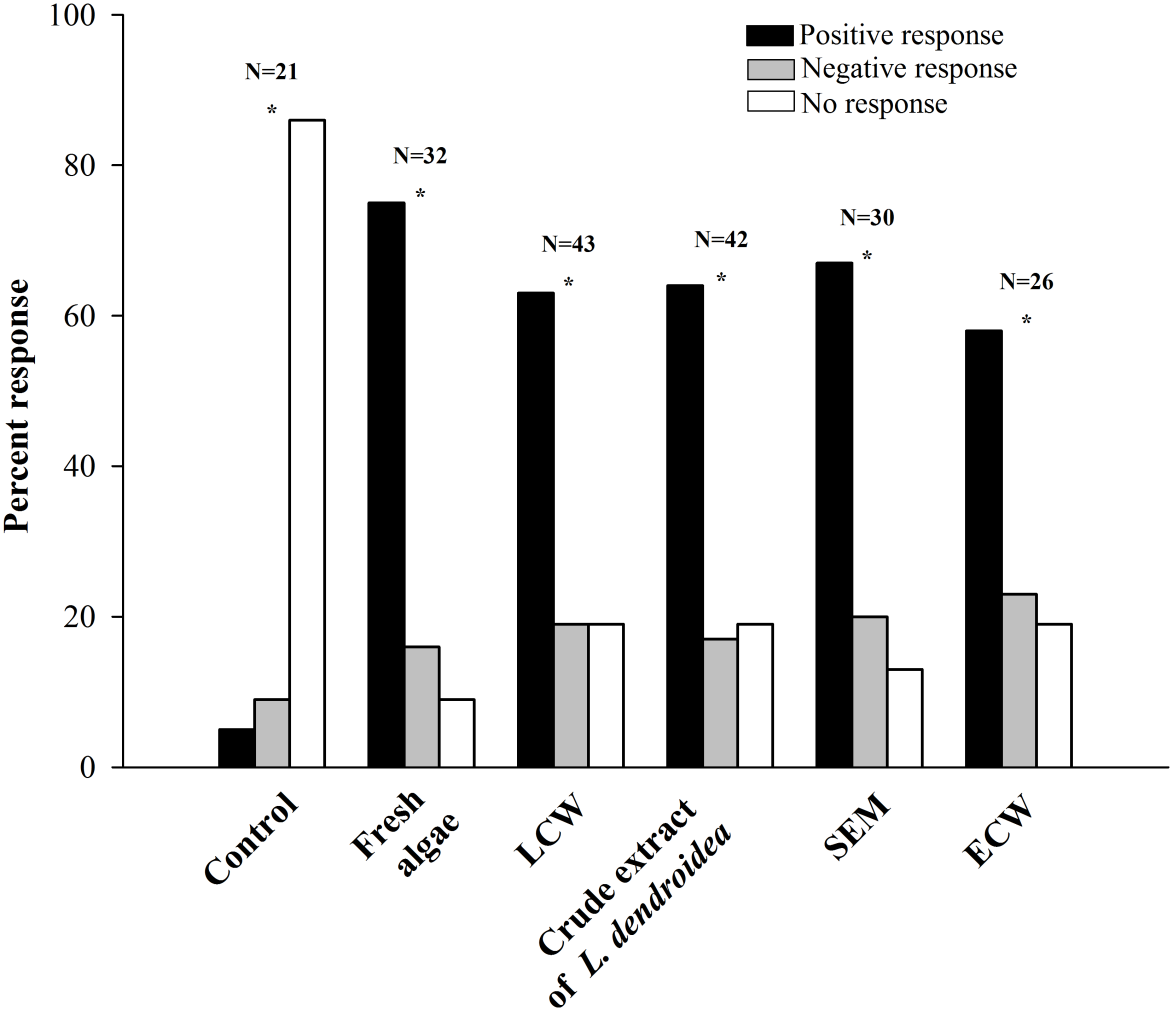
Percent responses of *A*. *brasiliana* specimens to different treatments in Y-maze bioassays. Control = artificial seawater without the presence of any *L*. *dendroidea* stimuli; LCW = *L*. *dendroidea* conditioned water; SEM = *Laurencia*-exuded metabolites in seawater extract; and ECW = elatol-conditioned water. Asterisks (*) denote significant differences between the response groups according to Chi-square tests (p ≤ 0.05).

Fig 4 shows that a significantly higher percentage of individuals of *A*. *brasiliana* (according to Chi-square tests) selected the arm of the Y-maze containing one type of chemical cue (Fig 4), represented by the presence of fresh *L*. *dendroidea* (p < 0.005; χ^2^ = 25.187; DF = 2), LCW (p < 0.005; χ^2^ = 16.791; DF = 2), *L*. *dendroidea* extract (p < 0.005; χ^2^ = 18.143; DF = 2), SEM (p < 0.05; χ^2^ = 15.20; DF = 2), or elatol-conditioned water (p < 0.005; χ^2^ = 7.00; DF = 2). These results are reinforced by the absence of preferential displacement (no response) of 86% of the 21 individuals of *A*. *brasiliana* in our control tests (p < 0.005; χ^2^ = 26.00; DF = 2).

## Discussion

Our results showed that the mollusc *Aplysia brasiliana* is able to detect and respond to chemicals produced and exuded by *Laurencia dendroidea*. This mollusc showed a positive chemotaxis by the presence of live seaweed, its water exudates in conditioned water (LCW) and also to its lipophilic extract (SEM).

Benthic marine invertebrates sense molecules from other organisms and use these compounds in different ecological contexts as communication, navigation and foraging behaviour [36]. These organisms produce and detect a wide range of polar and nonpolar compounds, but their identities are poorly understood [7]. Here, the lipophilic compounds exuded by *L*. *dendroidea* into seawater were detected by GC/MS analysis. Among them, the halogenated sesquiterpene (+)-elatol (**2**) was identified and revealed as one of the major compounds. In adition, the analysis of *L*. *dendroidea* extract revealed (+)-elatol as the major compound produced. These results indicated that (+)-elatol was exuded by alga into seawater.

The nonpolar (+)-elatol (**2**) is a known terpenoid isolated from *Laurencia* species that has multiple ecological functions, including anti-herbivory [12] and antifouling activities [37]. In our work, (+)-elatol attracted *A*. *brasiliana* in Y-maze experiments indicating its potencial as a chemical mediator in the interaction between *Laurencia* and *Aplysia*, its associated fauna.

Liposoluble terpenoids can mediate different alga-herbivore interactions. Halimedatetraacetate is a sesquiterpenoid produced by the green alga *Halimeda incrassata* identified as a foraging cue for the specialist gastropod *Elysia tuca* [38]. As one of the few herbivores that preferentially consume *Halimeda* species, *Elysia* may locate its prey by tracking *Halimeda*-derived chemical cues. This herbivore is frequently found associated to *H*. *incrassata*, which is able to sequester its chemical defenses to become unpalatable to its predators [39].

The oligophagous herbivorous molluscs belonging to the genus *Aplysia* feed primarily on red seaweeds and as well as the mentioned gastropod *Elysa tuca*, they are able to accumulate algal metabolites from these diets, which have been hypothesized to function as a defense against predators [13,16,18]. In fact, some of the halogenated sesquiterpenes isolated from *L*. *dendroidea*, such as the enantiomer of 10-bromo-9-hydroxy-chamigra-2,7(14)-diene (**1**), (+)-elatol (**2**), (*Z*)-10,15-dibromo-9-hydroxy-chamigra-1,3(15),7(14)-triene (**3**), (*E*)-10,15-dibromo-9-hydroxy-charmigra-1,3(15),7(14)-triene (**4**), isoobtusol (**5**), cartilagineol (**9**), obtusol (**10**) and rogiolol (**15**) have also been found in *Aplysia* species [16,18,26, 30, 40–43]. These findings reinforce the importance of these chemicals in the close relationship maintained between *Laurencia* and the sea hares belonging to the genus *Aplysia*.

Chemoreception can be the principal sensory system driving behavior for several marine organisms, including slowmoving molluscs belonging to the genus *Aplysia* [36]. Specific receptors involved in chemical signaling are able to detect and to bind to different compounds as water-soluble amino acids, organic acids or liposoluble terpenoids [44]. The sea hare *Aplysia californica*, for example, has a well-developed chemical sense with rhinophores and tentacles that help it to localize odors of its main food, i.e., seaweeds belonging to *Plocamium* and *Laurencia* genus [45]. *A*. *brasiliana*, which usually swims in order to find food [46], probably use similar sensors for localization. Our results provide evidence that secondary metabolites are important chemical cues mediating the interaction between *A*. *brasiliana* and *L*. *dendroidea*.

Identification of chemical communication and discrimination between evolved functions (signals) and unintentional releases (cues) are significant challenges in aquatic biology [8]. Chemical signals are intentionally released by the sender to convey information about presence of the signaler or the signaler’s environment changing the behavior of the receiver; this change of behavior must be profitable to sender and receiver. In general, signals must be honest and reliable. Already chemical cues are compounds unintentionally released by the sender that any other organism can use as a guide to display a particular behavior, benefiting the receiver exclusively [47] Distinguishing these two modes of information is essential in identifying, properly, a chemical substance that triggers a certain behavior in another individual, and only a closer look at the chemical compounds involved brings clarification about its ecological and evolutionary interactions [48]. Here, for the first time, the combination of experiments allowed to verify that the sea hare *A*. *brasiliana* is attracted by the sesquiterpene (+)-elatol as a chemical cue at different concentrations. In the experiments using conditioned water and pure elatol, the concentration of this compound was probably higher than that in natural conditions. On the other hand, in experiments using a unique piece of algal thallus, the exudated concentrations are expected to be similar to natural conditions, and the result obtained in this assay was similar that verified using conditioned water and pure elatol. In all cases, elatol present in the water was sufficient to attract *A*. *brasiliana*.

Additionaly, the diurnal production of elatol by *L*. *dendroidea* is dynamic and strongly correlated to the primary photosynthetic metabolism [49]. So, it is expected that its surface concentration is variable creating different scenarios for the chemical sinalization and interactions between the host seaweed and its associated fauna including the mollusc *A*. *brasiliana*.

Several species of *Laurencia* contain refractile membrane-bound vesicles (*corps en cerise*) in the outer cells or cortical layer, which act as locations for the biosynthesis and storage of halogenated secondary metabolites [50], including elatol. Through a transport system, these metabolites reach the surface and provide essential cues as surface-mediated chemicals [51]. A previous study revealed that elatol occurs in very low amounts on the surface of *L*. *dendroidea* (0.006 mg g^-1^) when compared to the highest within-thallus quantities (9.89 mg g^-1^) based on algal dry weight [52]. Low concentrations of elatol on the surface of *L*. *dendroidea* did not inhibit herbivory by sea urchins, settlement of barnacle larvae, or mussel attachment [51], but is probably enough to attract *A*. *brasiliana*.

Compared to adults, juvenile sea hares are known to be subject to high predation pressure [53]. In addition, juvenile sea hares have restricted diets, often eating only one or a few types of red seaweeds [54]. Several small sedentary herbivores (mesograzers such as amphipods, crabs and polychaetes) can significantly decrease their susceptibility to predation by living among and feeding on seaweeds that are avoided by predators [55]. In our bioassays, we only used juvenile *A*. *brasiliana* that were found associated with *L*. *dendroidea* in the field. Thus, young individuals of *A*. *brasiliana* are mesograzers that use *L*. *dendroidea* both food and as a chemical source that protects from predators.

In conclusion, our discovery of *Aplysia* foraging cues reveals that this mollusc uses prey secondary metabolites as foraging cues. The sesquterpene (+)-elatol can facilitate the encounter of *A*. *brasiliana* with *L*. *dendroidea*, but it is still not known if other compounds are involved in this process. Thus, elatol represents for this alga both an evolved function against natural enemies such as pressure by conusmers [12] and fouling [37], and also a cue that benefits the sea hare *A*. *brasiliana*.

## Supporting information

S1 Table NMR data and all the MS and NMR spectra of the isolated compounds 1–5 found in *L*. *dendroidea*. (DOC)
