# Supplementary material for: Detection of a chemical cue from the host seaweed Laurencia dendroidea by the associated mollusc Aplysia brasiliana
Source: PLoS One. 2017 Nov 2;12(11):e0187126. doi: 10.1371/journal.pone.0187126 (PMC5667859; doi:10.1371/journal.pone.0187126)
Supplement: S1 Table — (DOC) [file pone.0187126.s001.doc]

**SUPPORTING INFORMATION**

NMR data and all the MS and NMR spectra of the isolated compounds 1–5

**Detection of a chemical cue from the host seaweed *Laurencia dendroidea* by the associated mollusc *Aplysia brasiliana***

N. Nocchi^1,2^, A. R. Soares^2*^, M. L. Souto^3^, J. J. Fernández^3^, M. N. Martin^3^ & R. C. Pereira^1,4*^

^1^Programa de Pós-graduação em Dinâmica do Oceano e da Terra, Universidade Federal Fluminense, Avenida General Milton Tavares de Souza, Campus da Praia Vermelha, 24210-346, Niterói, Brazil.

^2^Grupo de Produtos Narturais de Organismos Aquáticos (GPNOA), Universidade Federal do Rio de Janeiro, Núcleo em Ecologia e Desenvolvimento Sócio-Ambiental de Macaé, Avenida São José do Barreto, 764, 27971-550, Macaé, Brazil.

^3^Instituto Universitario de Bio-Orgánica “Antonio González” (IUBO), Centro de Investigaciones Biomédicas de Canarias (CIBICAN), Departamento de Química Orgánica, Universidad de La Laguna (ULL), Avda. Astrofísico Francisco Sánchez, 2, 38206 La Laguna, Tenerife, España.

^4^Current Address: Instituto de Pesquisas Jardim Botânico do Rio de Janeiro, CEP 22460-330, Rio de Janeiro, Brazil.

*Correspondence and requests for materials should be addressed to A.R.S, and R.C.P. (email: [angelica.r.soares@gmail.com](mailto:angelica.r.soares@gmail.com) and

rcrespo@id.uff.br)

**S1 Table. TABELA III. 1**. NMR data of 10-bromo-9-hydroxy-chamigra-2,7(14)-diene **(1)**, (+)-elatol **(2)**, (*Z*)-10,15-dibromo-9-hydroxy-chamigra-1,3(15),7(14)-triene **(3)**, (*E*)-10,15-dibromo-9-hydroxy-chamigra-1,3(15),7(14)-triene **(4)**  and isoobtusol **(5).** = chemical shift in ppm; m = multiplicity; *J* = coupling constant in Hertz.

|  |  | | | |  | | | |  | | | |  | | | |  | | | |
| --- | --- | --- | --- | --- | --- | --- | --- | --- | --- | --- | --- | --- | --- | --- | --- | --- | --- | --- | --- | --- |
|  |  | | | |  | | | |  | | | |  | | | |  | | | |
|  |  | | | |  | | | |  | | | |  | | | |  | | | |
|  |  | | | |  | | | |  | | | |  | | | |  | | | |
|  | **(1)** | | | | **(2)** | | | | **(3)** | | | | **(4)** | | | | **(5)** | | | |
| **nº C** | **δ ^13^C** | **δ ^1^H** | **m** | ***J* (Hz)** | **δ ^13^C** | **δ ^1^H** | **M** | ***J* (Hz)** | **δ ^13^C** | **δ ^1^H** | **m** | ***J* (Hz)** | **δ ^13^C** | **δ ^1^H** | **m** | ***J* (Hz)** | **δ ^13^C** | **δ ^1^H** | **m** | ***J* (Hz)** |
| **1** | 29.96 | 2.22 | d | 17,5 | 38.76 | 2.35 | d | 17.4 | 129.22 | 6.21 | d | 10.3 | 126.98 | 6.66 | d | 10.5 | 33.9 | 2.80 | dd | 15.6 - 3.2 |
|  |  | 2.11 | d | 19,0 |  | 2.57 | d | 17.6 |  |  |  |  |  |  |  |  |  | 3.09 | d | 14.5 |
| **2** | 119.61 | 5.28 | s |  | 124.2 | - |  |  | 132.76 | 5.89 | d | 10.2 | 136.46 | 6.11 | d br | 10.5 | 65.2 | 4.44 | S |  |
| **3** | 132.5 | - |  |  | 128.22 | - |  |  | 139.5 | - |  |  | 137.2 | - |  |  | 71.1 | - |  |  |
| **4** | 27.38 | 1.81 | m |  | 29.48 | 1.81 | d | 11.8 | 25.65 | 1.99 | m |  | 27.5 | 2.32 | m |  | 33.2 | 1.84 | dd | 14.8 – 2.0 |
|  |  | 1.62 | m |  |  | 1.94 | d | 16.4 |  | 1.75 | ddd | 18.1 - 10.8 - 3.8 |  | 2.20 | m |  |  | 2.24 | t | 14.3 |
| **5** | 25.48 | 1.82 | m |  | 25.75 | 1.61 | m |  | 24.38 | 2.64 | m |  | 26.25 | 1.95 | m |  | 25.6 | 1.78 | dd | 14.2 – 2.7 |
|  |  | 1.67 | m |  |  | 1.81 | d | 11.8 |  |  |  |  |  | 1.78 | m |  |  | 2.04 | dt | 13.8 – 3.3 |
| **6** | 47 | - |  |  | 49.3 | - |  |  | 51.8 | - |  |  | 52 | - |  |  | 44 | - |  |  |
| **7** | 141.1 | - |  |  | 140.9 | - |  |  | 143 | - |  |  | 143.4 | - |  |  | 148.1 | - |  |  |
| **8** | 37.77 | 2.70 | d | 14,13 | 38.1 | 2.49 | d | 14.5 | 37.98 | 2.71 | dd | 15.9 – 2.1 | 37.89 | 2.73 | dd | 15.1-2.2 | 39.4 | 2.43 | d | 10.6 |
|  |  | 2.48 | dd | 14,5- 2,4 |  | 2.62 | d | 14.6 |  | 2.59 | dd | 15.0 -2.4 |  | 2.61 | dd | 15.1-2.3 |  | 2.7 | t | 12.1 |
| **9** | 72.23 | 4.16 | s |  | 72.33 | 4.14 | d | 2.3 | 71.96 | 4.17 | m |  | 71.98 | 4.19 | m |  | 69.8 | 3.66 | d | 3.1 |
| **10** | 71.61 | 4.69 | d | 2,7 | 71.03 | 4.60 | d | 2.3 | 70.32 | 4.65 | d | 2.9 | 70.24 | 4.63 | d | 2.9 | 76.2 | 4.41 | d | 12.4 |
| **11** | 43.1 | - |  |  | 43.27 | - |  |  | 42.7 | - |  |  | 42.8 | - |  |  | 43.7 | - |  |  |
| **12** | 20.33 | 1.04 | s |  | 20.9 | 1.05 | s |  | 21.45 | 1.24 | s |  | 21.54 | 1.27 | s |  | 25.6 | 1.05 | s |  |
| **13** | 24.11 | 1.05 | s |  | 24.3 | 1.06 | s |  | 26.57 | 1.01 | s |  | 26.67 | 1.03 | s |  | 24.9 | 1.34 | s |  |
| **14** | 115.79 | 4.81 | s |  | 116.07 | 4.78 | s |  | 117.36 | 4.82 | s |  | 117.95 | 4.86 | s |  | 113.83 | 4.92 | s |  |
|  |  | 5.09 | s |  |  | 5.11 | s |  |  | 5.10 | s |  |  | 5.16 | s |  |  | 5.17 | s |  |
| **15** | 22.84 | 1.58 | s |  | 19.59 | 1.69 | s |  | 105.39 | 6.11 | s |  | 101.51 | 5.93 | s |  | 32.8 | 1.92 | s |  |

**S1 Fig. Mass spectra obtained by electron impact (EI-MS, 70eV)** **of 10-bromo-9-hydroxy-chamigra-2,7(14)-diene (1)**

**
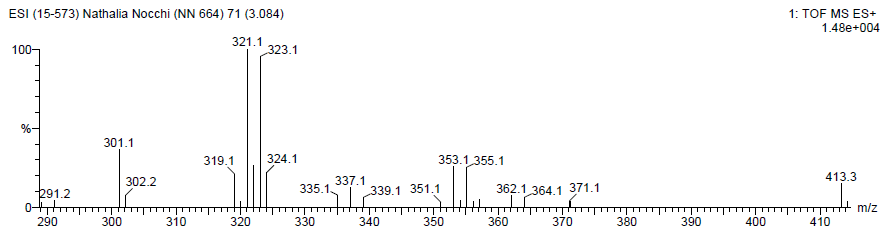
**

**S2 Fig. Mass spectra obtained by electrospray (ESI-MS) of 10-bromo-9-hydroxy-chamigra-2,7(14)-diene (1) (M+Na)**

**
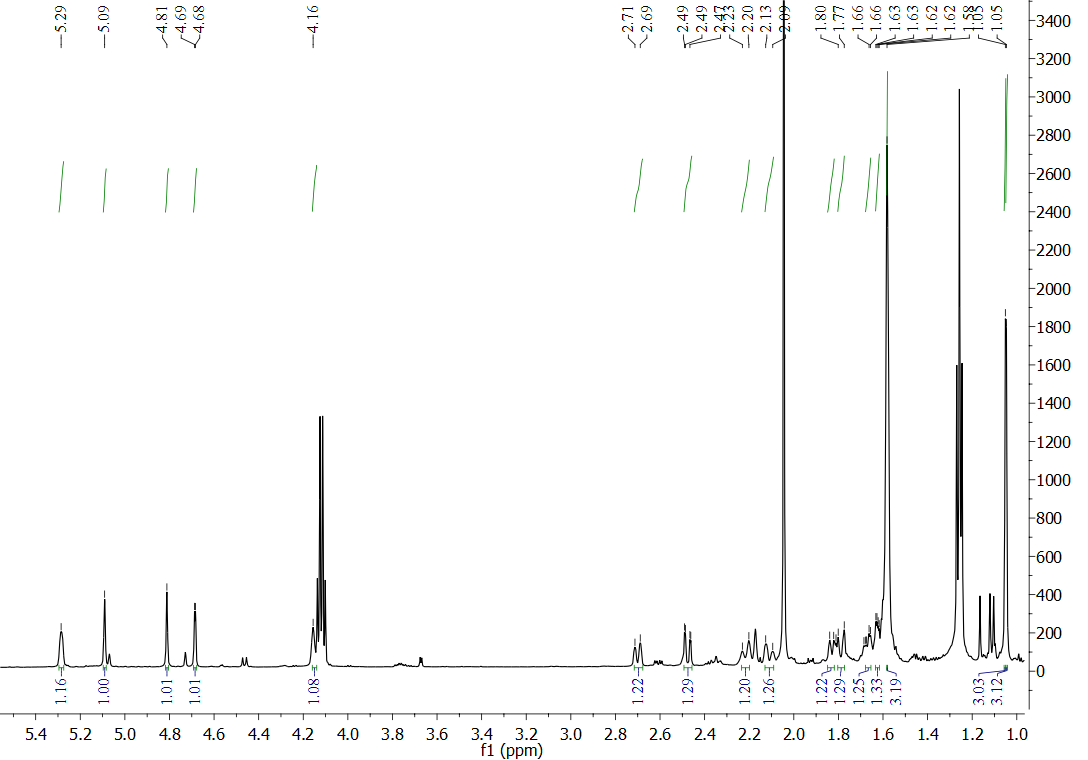
**

**S3 Fig. ^1^H NMR spectra (600 MHz, CDCl3) of 10-bromo-9-hydroxy-chamigra-2,7(14)-diene (1)**


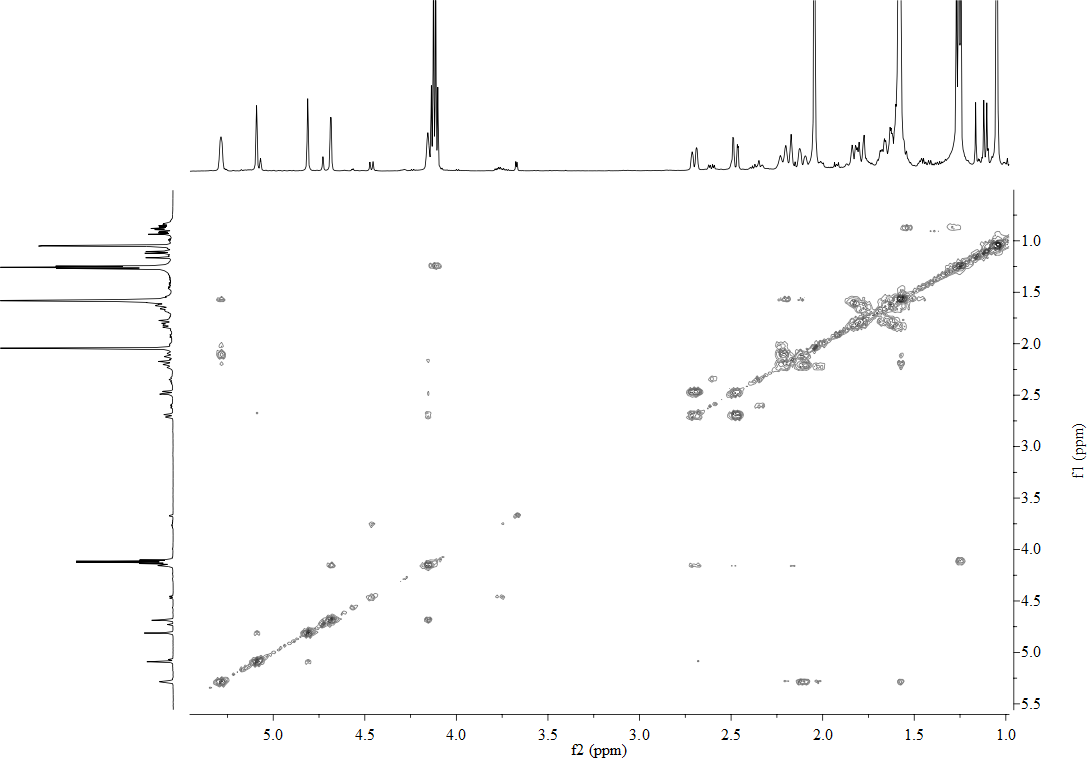


**S4 Fig. COSY NMR spectra (600 MHz, CDCl3) of 10-bromo-9-hydroxy-chamigra-2,7(14)-diene (1)**


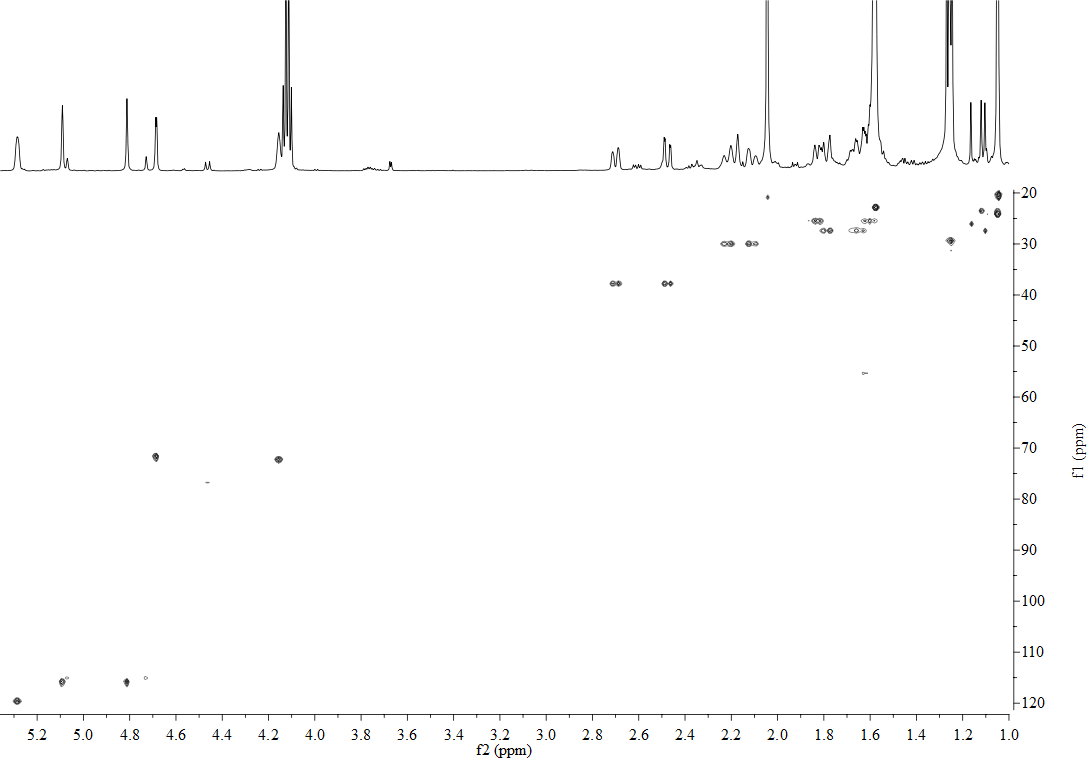


**S5 Fig. HSQC NMR spectra (600 MHz, CDCl3) of 10-bromo-9-hydroxy-chamigra-2,7(14)-diene (1)**

**S6 Fig. Mass spectra obtained by electron impact (EI-MS, 70eV) of (+)-elatol (2)**

**
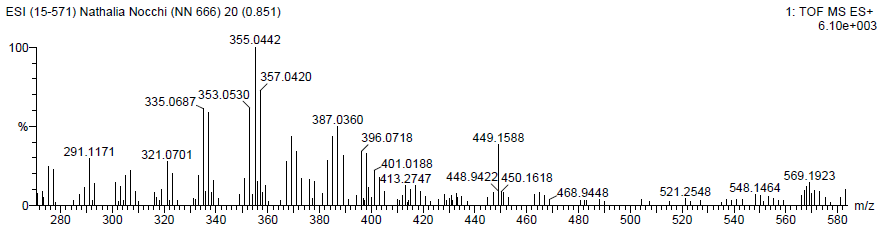
**

**S7 Fig. Mass spectra obtained by electrospray (ESI-MS) of (+)-elatol (2) (M+Na)**


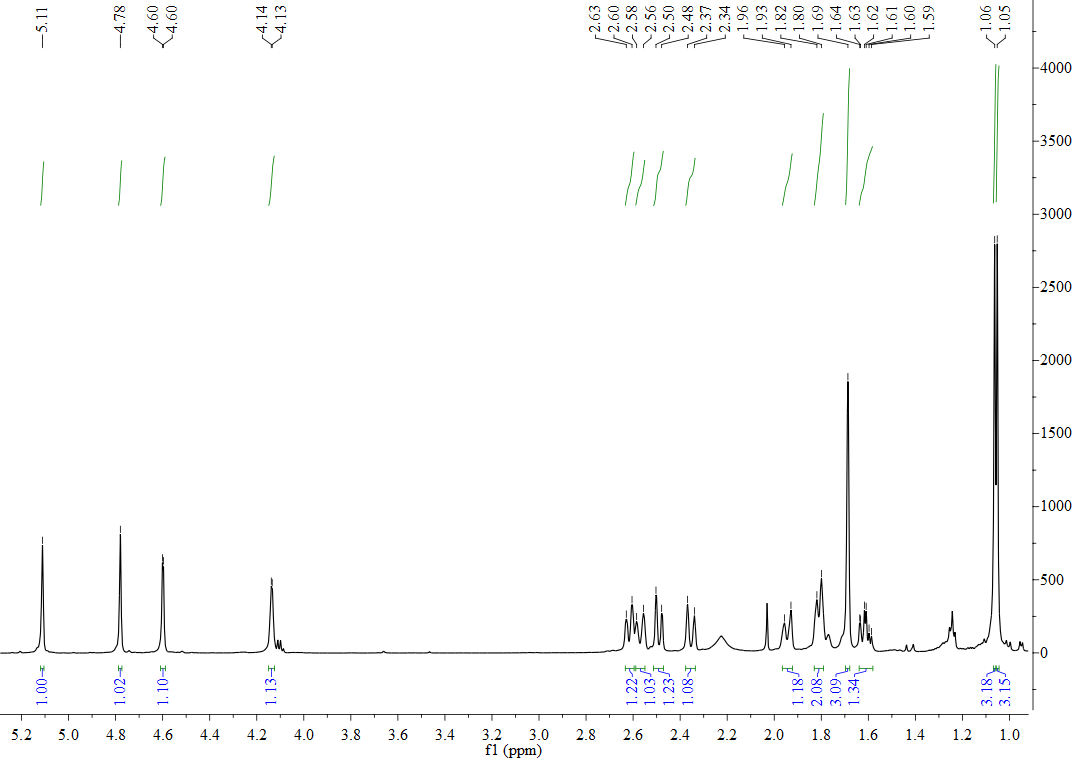


**S8 Fig. ^1^H NMR spectra (600 MHz, CDCl3) of (+)-elatol (2)**


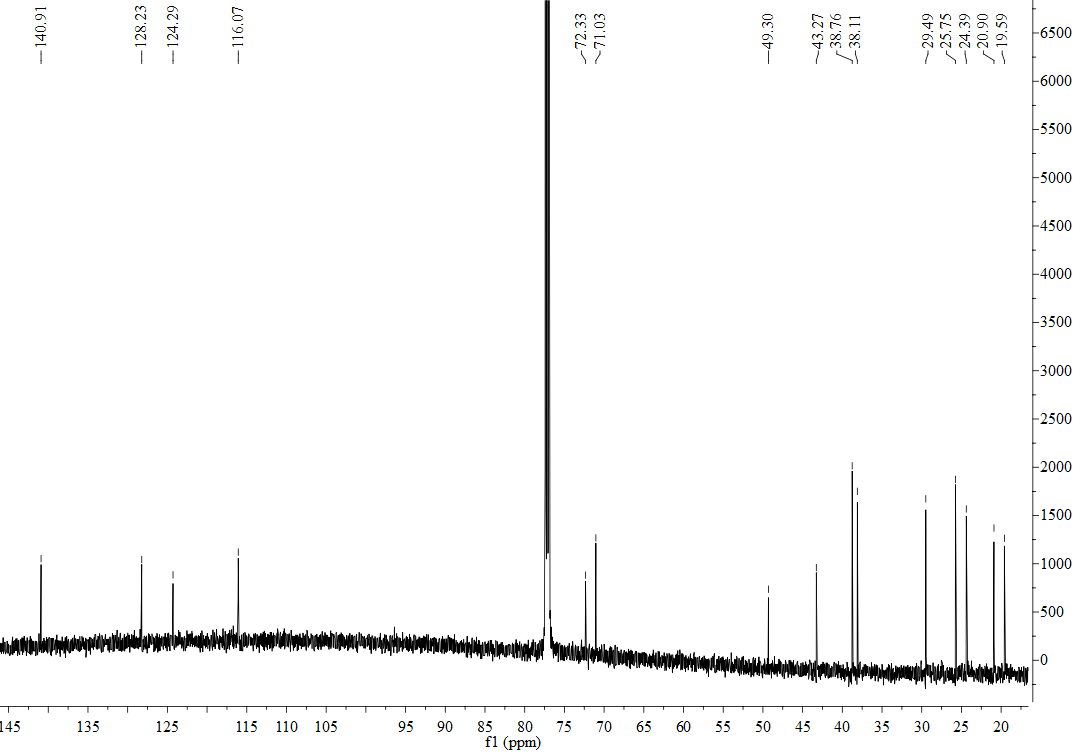


**S9 Fig. ^13^C NMR spectra (600 MHz, CDCl3) of (+)-elatol (2)**


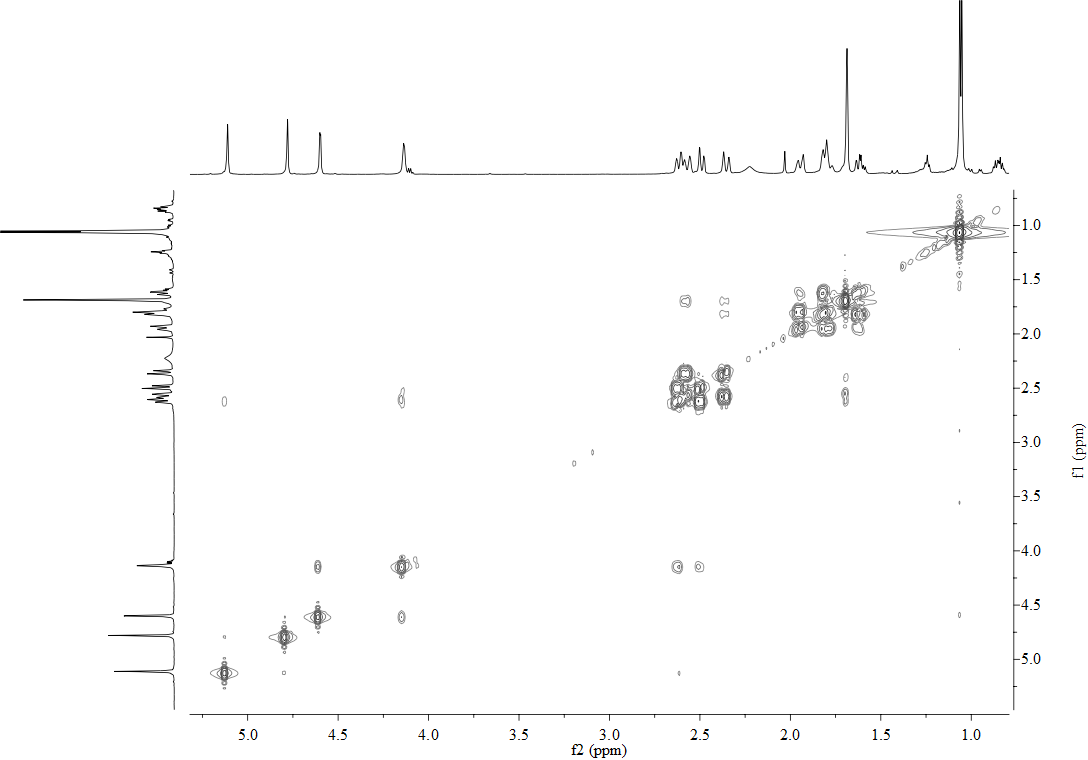


**S10 Fig. COSY NMR spectra (600 MHz, CDCl3) of (+)-elatol (2)**


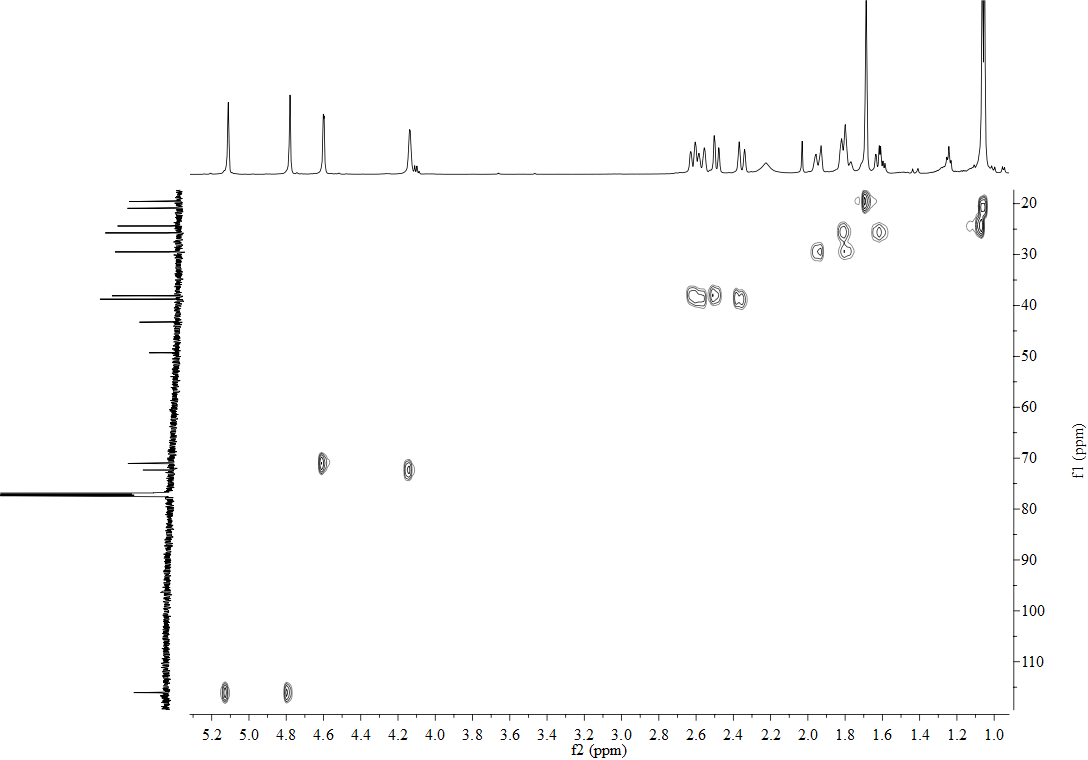


**S11 Fig. HSQC NMR spectra (600 MHz, CDCl3) of (+)-elatol (2)**

**
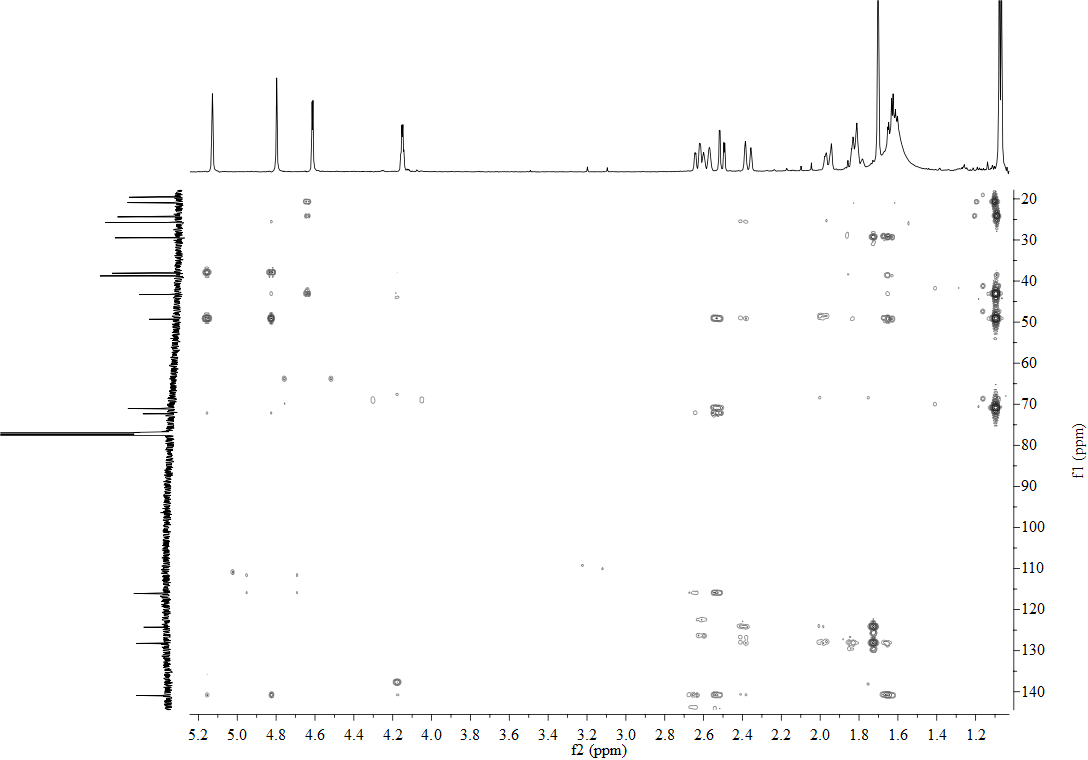
**

**S12 Fig. HMBC NMR spectra (600 MHz, CDCl3) of (+)-elatol (2)**

**S13 Fig. Mass spectra obtained by electron impact (EI-MS, 70eV) of** **(*Z*)-10,15-dibromo-9-hydroxy-chamigra-1,3(15),7(14)-triene (3)**

**
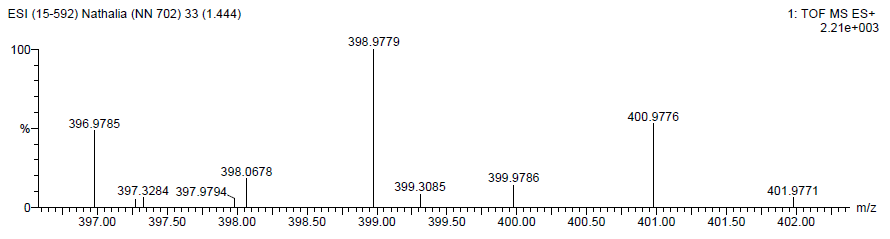
**

**S14 Fig. Mass spectra obtained by electrospray (ESI-MS) of** **(*Z*)-10,15-dibromo-9-hydroxy-chamigra-1,3(15),7(14)-triene (3) (M+Na)**

**
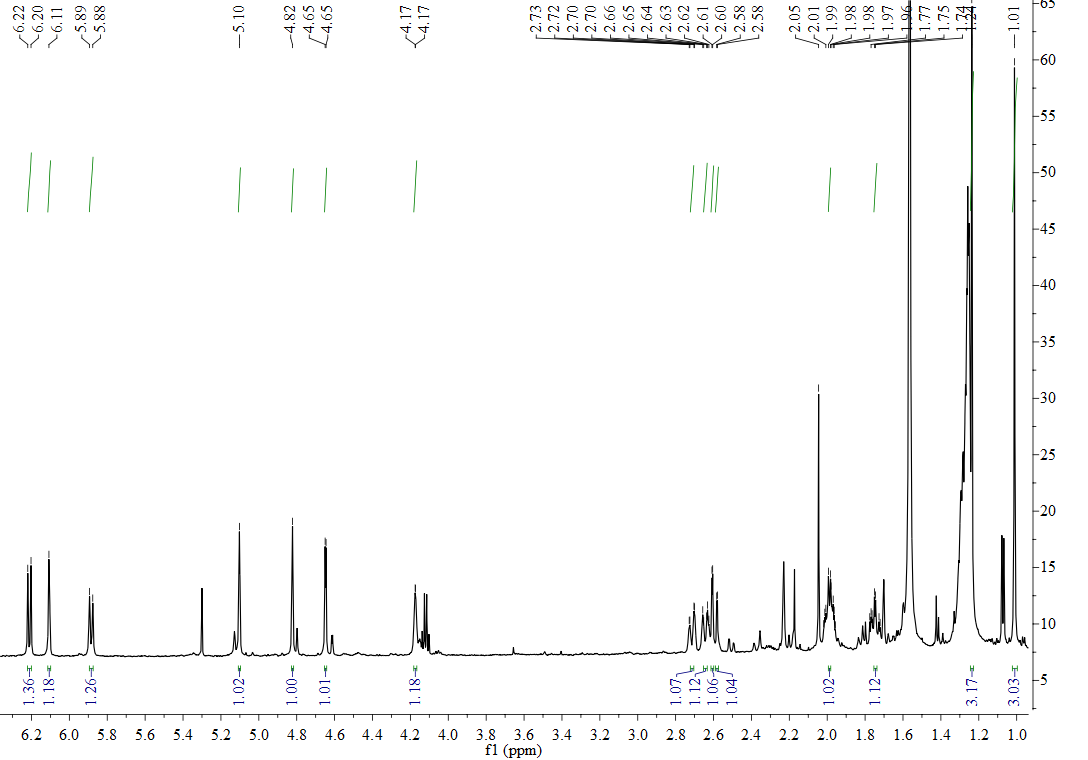
**

**S15 Fig. ^1^H NMR spectra (600 MHz, CDCl3) of (*Z*)-10,15-dibromo-9-hydroxy-chamigra-1,3(15),7(14)-triene (3)**

**
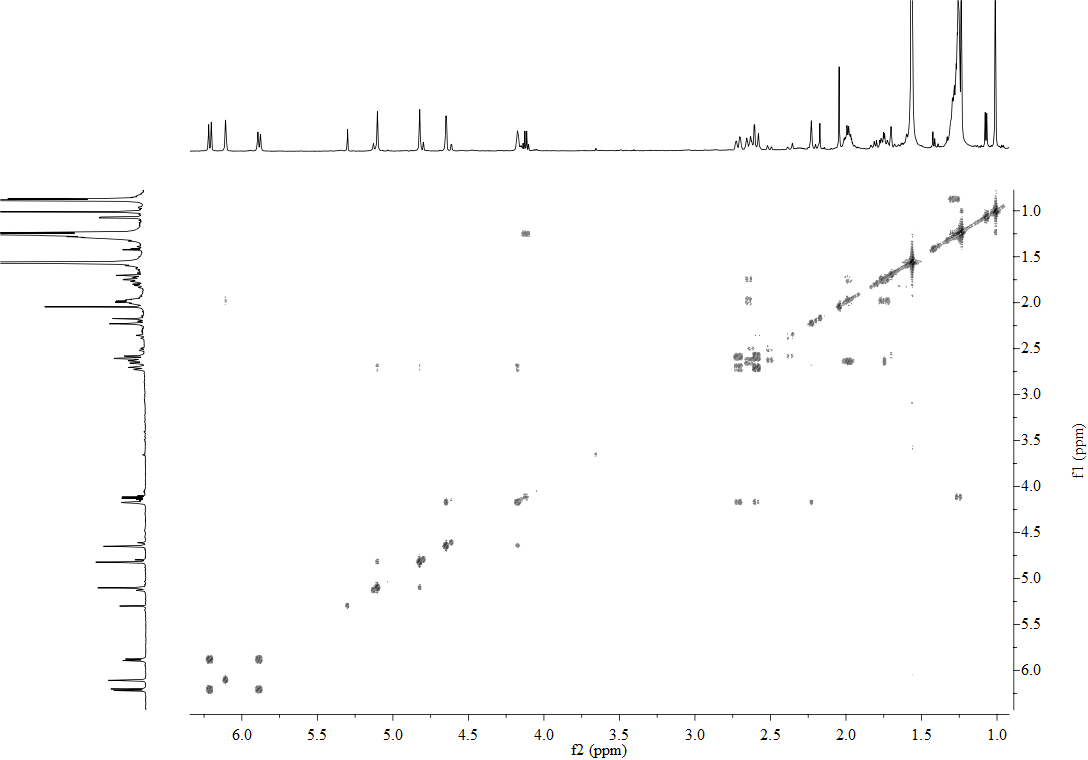
**

**S16 Fig. COSY NMR spectra (600 MHz, CDCl3) of (*Z*)-10,15-dibromo-9-hydroxy-chamigra-1,3(15),7(14)-triene (3)**

**
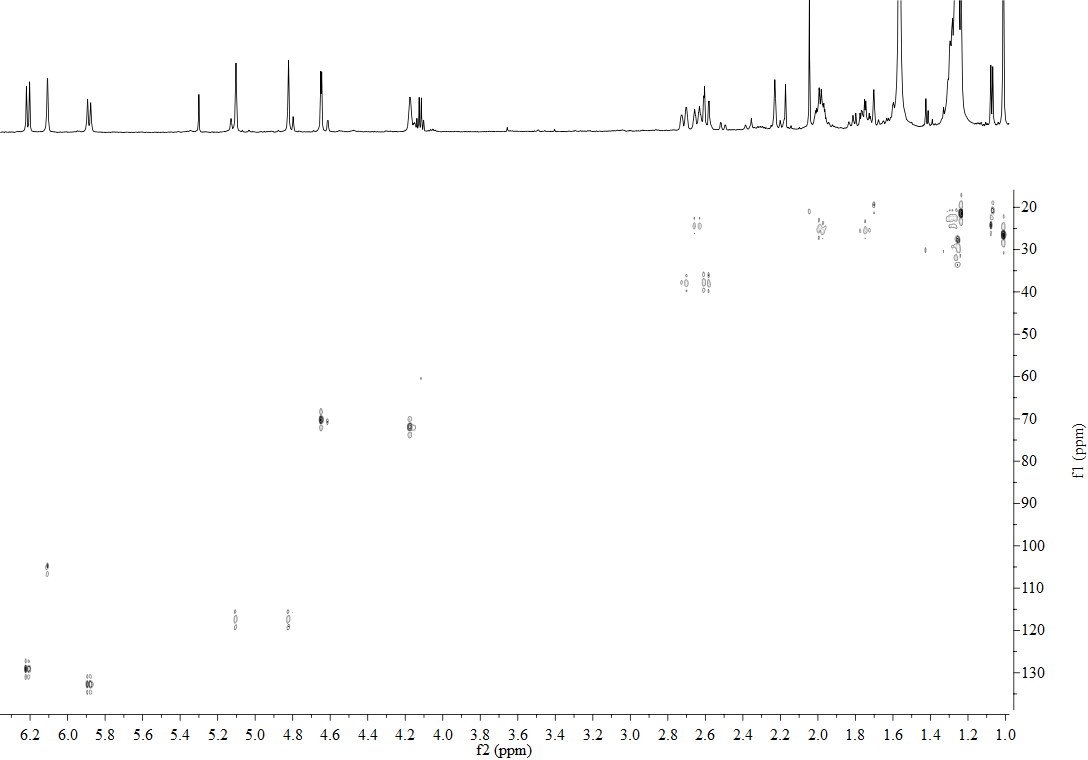
**

**S17 Fig. HSQC NMR spectra (600 MHz, CDCl3) of (*Z*)-10,15-dibromo-9-hydroxy-chamigra-1,3(15),7(14)-triene (3)**

**S18 Fig. Mass spectra obtained by electron impact (EI-MS, 70eV) of** **(*E*)-10,15-dibromo-9-hydroxy-chamigra-1,3(15),7(14)-triene (4)**


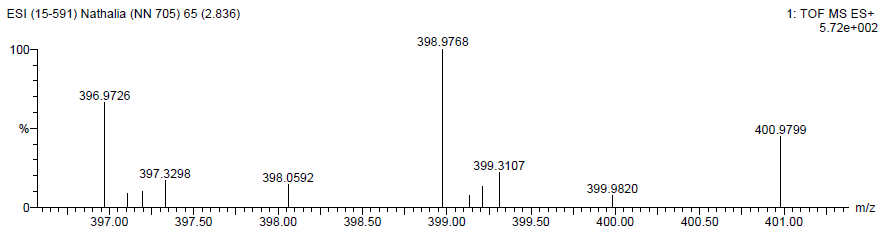


**S19 Fig. Mass spectra obtained by electrospray (ESI-MS) of** **(*E*)-10,15-dibromo-9-hydroxy-chamigra-1,3(15),7(14)-triene (4)** **(M+Na)**

**
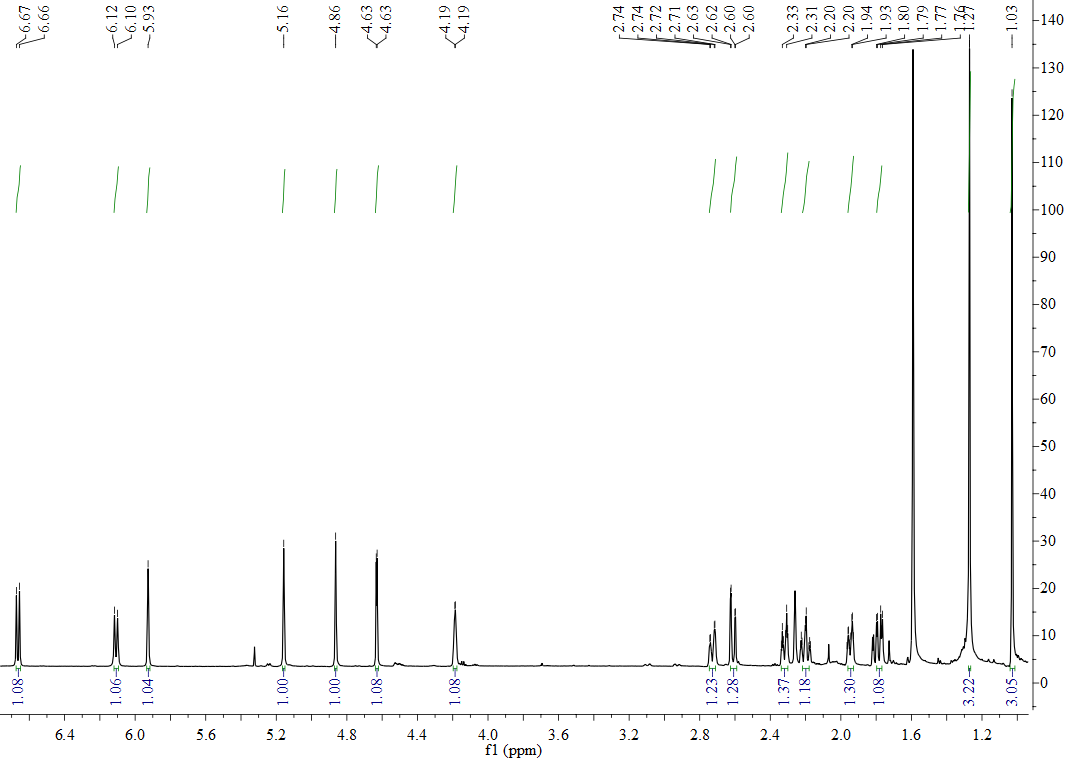
**

**S20 Fig. ^1^H** **NMR spectra (600 MHz, CDCl3) of (*E*)-10,15-dibromo-9-hydroxy-chamigra-1,3(15),7(14)-triene (4)**

**
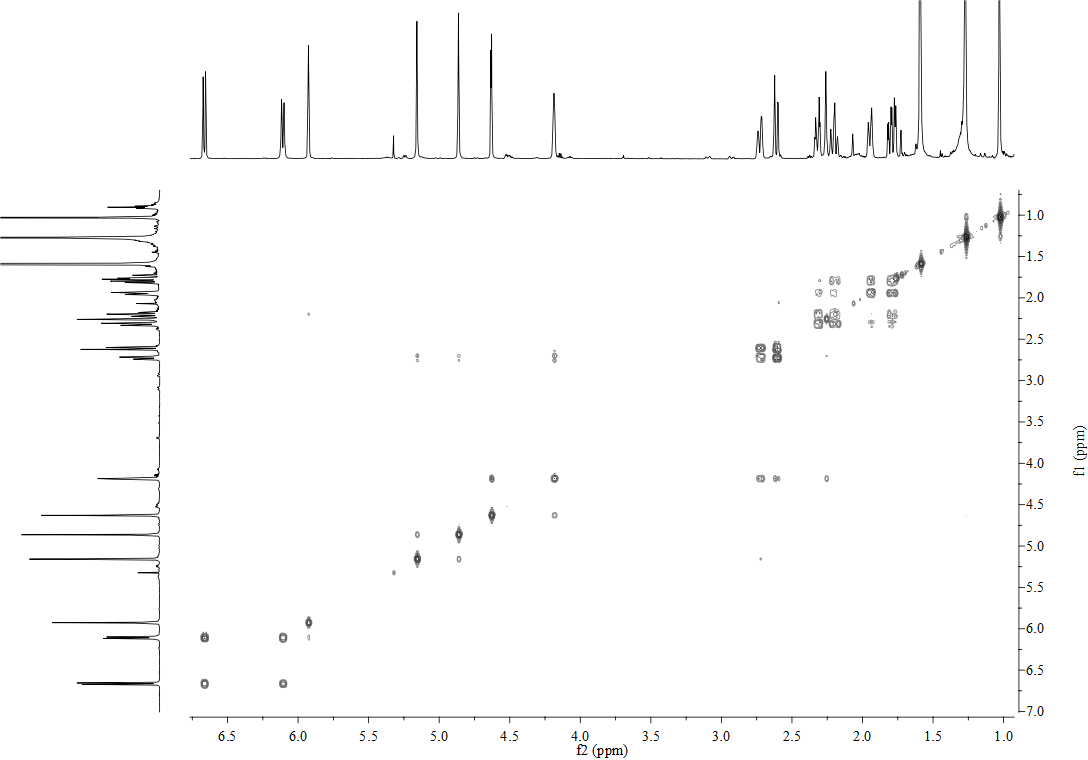
**

**S21 Fig. COSY NMR spectra (600 MHz, CDCl3) of (*E*)-10,15-dibromo-9-hydroxy-chamigra-1,3(15),7(14)-triene (4)**

**
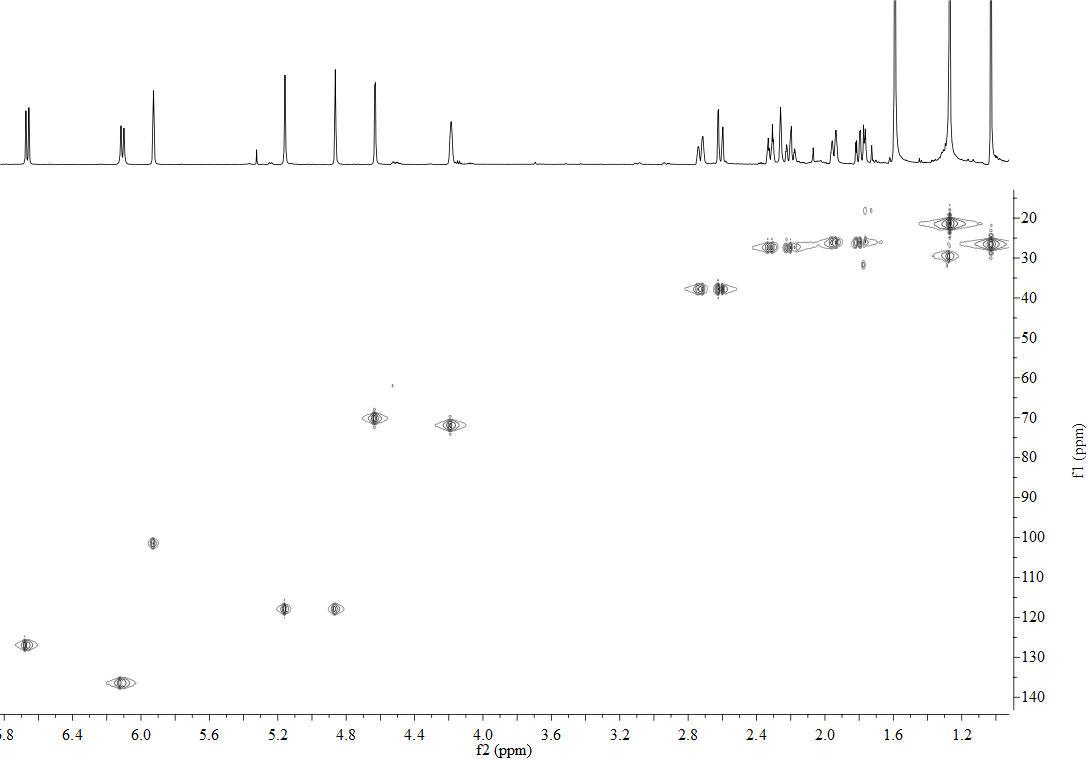
**

**S22 Fig. HSQC NMR spectra (600 MHz, CDCl3) of (*E*)-10,15-dibromo-9-hydroxy-chamigra-1,3(15),7(14)-triene (4)**

**S23 Fig. Mass spectra obtained by electron impact (EI-MS, 70eV) of** **isoobtusol (5)**

**
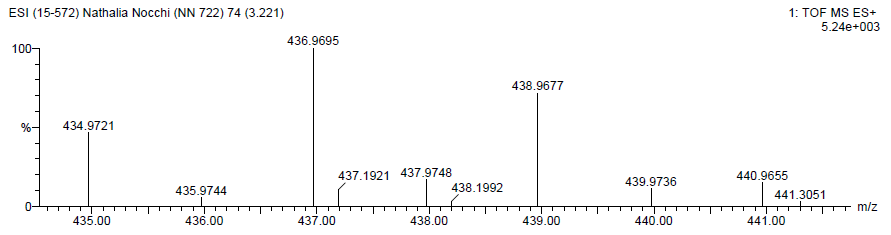
**

**S24 Fig. Mass spectra obtained by electrospray (ESI-MS) of** **isobtusol (5)** **(M+Na)**

**
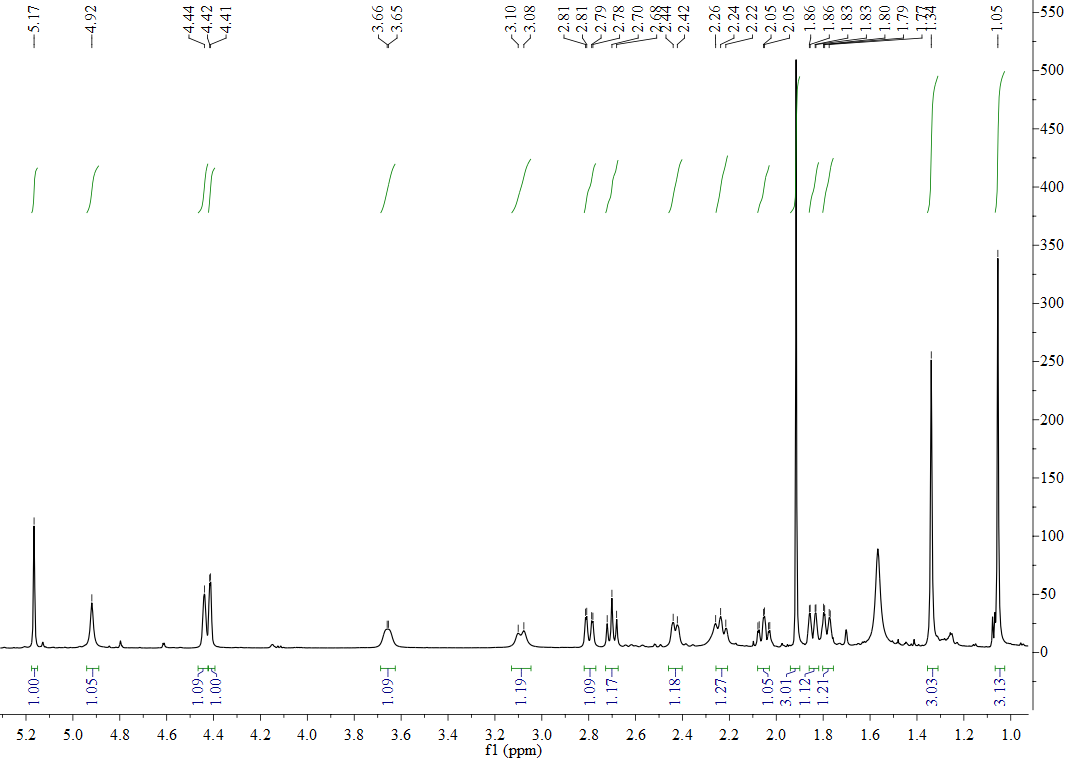
**

**S25 Fig. ^1^H NMR spectra (600 MHz, CDCl3) of isoobtusol (5)**

**
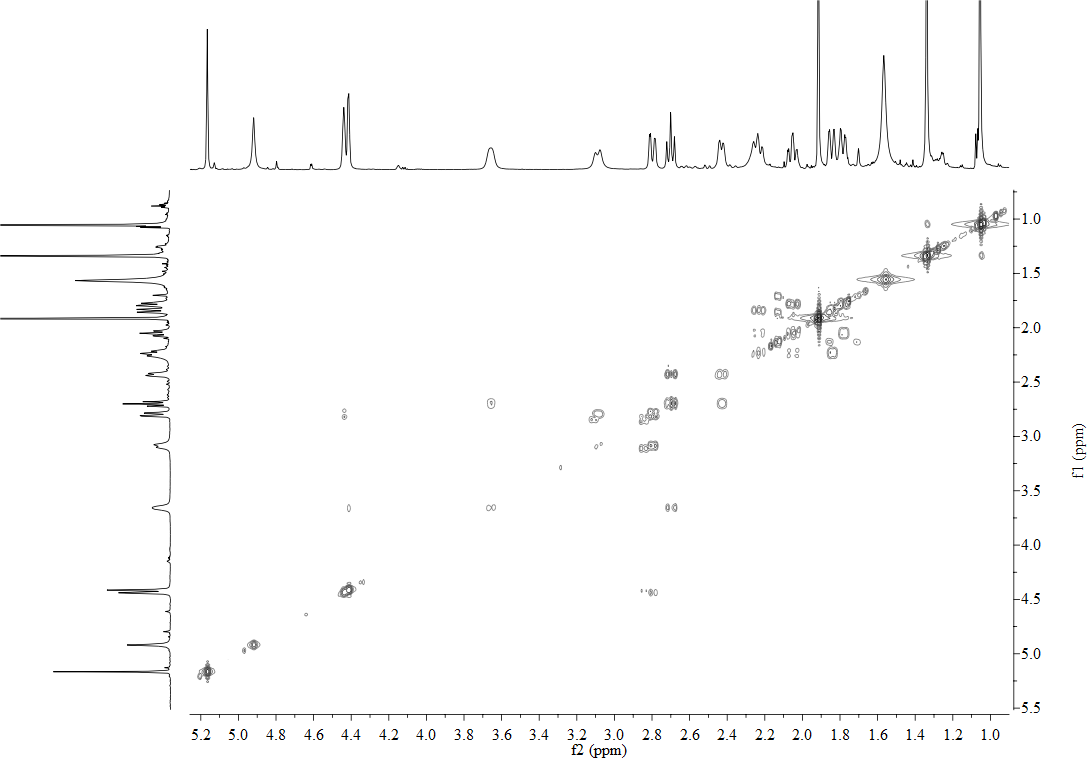
**

**S26 Fig. COSY NMR spectra (600 MHz, CDCl3) of isoobtusol (5)**

**
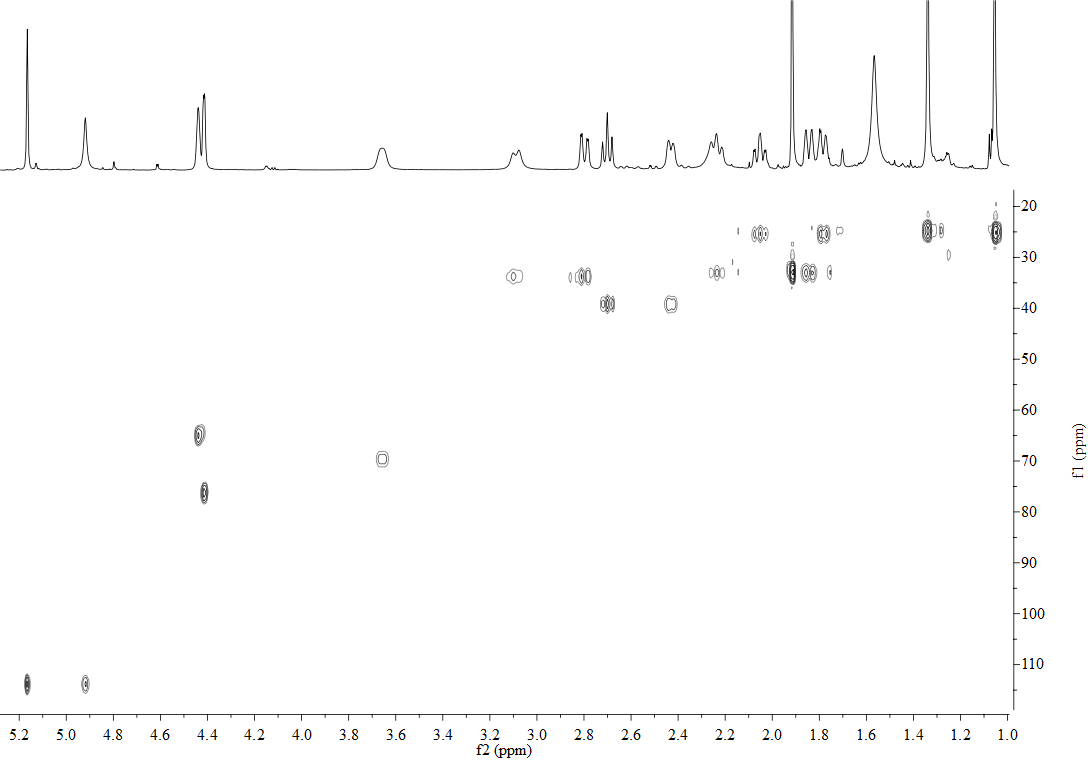
**

**S27 Fig. HSQC NMR spectra (600 MHz, CDCl3) of isoobtusol (5)**


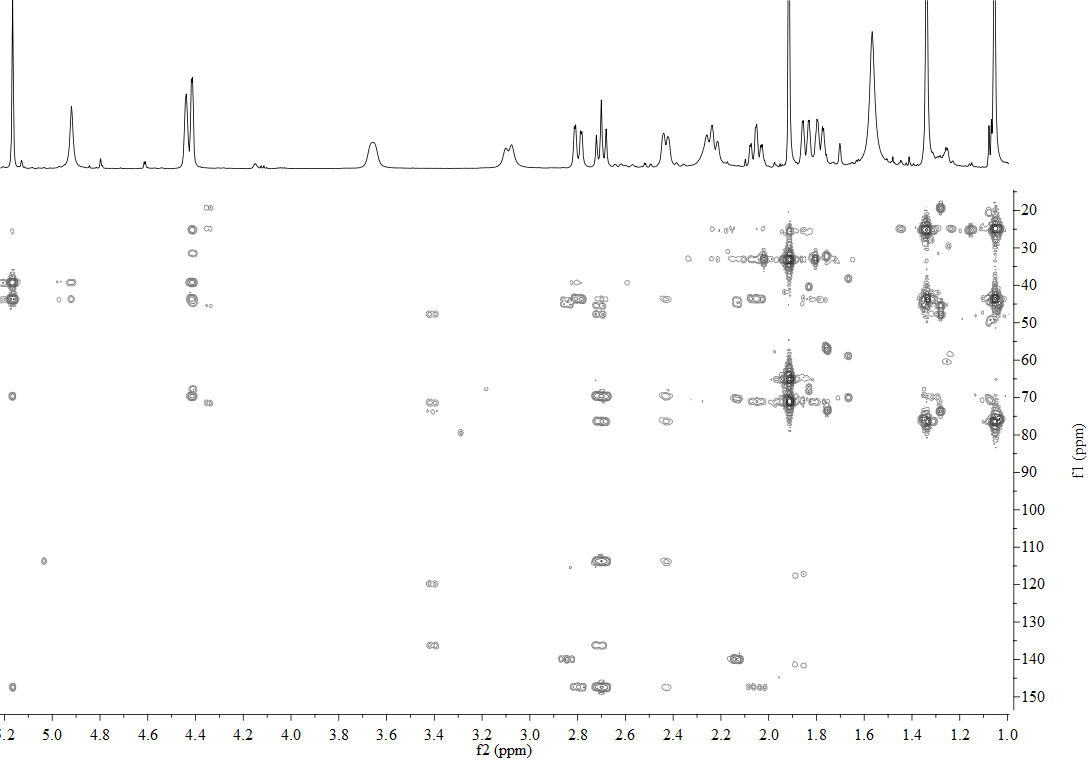


**S28 Fig. HMBC NMR spectra (600 MHz, CDCl3) of isoobtusol (5)**
